# FluEvoFormer: A Structure-Guided Generative Foundation Model for Prospective Influenza Antigenic Evolution and Vaccine Strain Selection

**DOI:** 10.3390/v18080843

**Published:** 2026-08-01

**Authors:** Pankaj Agarwal, Sumendra Yogarayan, Md. Shohel Sayeed

**Affiliations:** 1 Centre for Intelligent Cloud Computing, COE for Advanced Cloud, Multimedia University, Melaka 75450, Malaysia; pankaj.agarwal@krmangalam.edu.in (P.A.); shohel.sayeed@mmu.edu.my (M.S.S.); 2 School of Engineering & Technology, K.R. Mangalam University, Sohna Road, Gurugram 122103, Haryana, India; 3 Faculty of Information Science and Technology, Multimedia University (MMU), Melaka 75450, Malaysia

**Keywords:** influenza vaccine strain selection, antigenic evolution, hemagglutinin, neuraminidase, protein language model, graph transformer, generative AI, vaccine candidate ranking

## Abstract

Seasonal influenza vaccine strain selection remains challenging because circulating viruses may drift after vaccine recommendations are made. This study presents FluEvoFormer, a structure-guided generative foundation model for prospective influenza antigenic evolution forecasting and vaccine strain ranking. The framework jointly encodes hemagglutinin and neuraminidase sequences and incorporates residue-contact graphs. Separate prediction heads estimate future viral dominance and the vaccine–virus antigenic match. Controlled future-like variant stress testing, uncertainty adjustments, and clade balancing are then used to rank vaccine candidates. A rolling retrospective evaluation was performed for target seasons involving the influenza A(H1N1)pdm09 virus and influenza A(H3N2) virus. The evaluation used cutoff-restricted sequence records, hemagglutination inhibition data, vaccine-composition records, protein-structure resources, and vaccine-effectiveness indicators. The historical training corpus for the influenza A(H1N1) virus also contained pre-2009 seasonal records. FluEvoFormer achieved the lowest held-out antigenicity prediction error, with mean absolute error (MAE) values of 0.389 for the combined historical influenza A(H1N1) virus corpus and 0.456 for the influenza A(H3N2) virus corpus. It also improved the future dominance prediction, with Kullback–Leibler (KL) divergence values of 0.255 and 0.289, respectively. The model selected candidates with higher empirical normalized coverage scores in seven out of 10 influenza A(H1N1)pdm09 virus seasons and nine out of 10 influenza A(H3N2) virus seasons. The predicted coverage score showed a strong positive correlation with external vaccine-effectiveness estimates. These findings support FluEvoFormer as a computational decision-support framework for prioritizing influenza vaccine candidates before downstream laboratory and public health evaluations.

## 1. Introduction

Seasonal influenza remains a major public health challenge because circulating viruses continually evolve through genetic mutations and reassortment. Some of these genetic changes alter antibody recognition and produce antigenic drift. Vaccination reduces influenza-related illness, hospitalization, and severe outcomes, but the vaccine effectiveness varies among seasons, viral subtypes, age groups, vaccine platforms, and population immune histories [1,2,3]. One important cause of reduced protection is antigenic mismatch between the vaccine strain and the viruses that subsequently dominate the target season [4,5,6].

Influenza vaccine strains must be selected several months before the corresponding season, when the future circulating viral population is still uncertain [7]. The current strain-selection processes integrate global surveillance, genetic and antigenic characterization, epidemiological evidence, vaccine production considerations, and expert assessments. Computational forecasting can complement this process by estimating two quantities before the season begins: which viral lineages are most likely to expand and how closely candidate vaccine strains are expected to match those future viruses. Such models should support, rather than replace, laboratory antigenic characterization and public health decision making.

Hemagglutinin (HA) is the principal target of neutralizing antibodies and remains central to influenza vaccine strain assessments [8,9]. However, neuraminidase (NA) also contributes to viral release, replication fitness, and immune protection, and NA-directed antibodies can provide protection independently of HA-directed responses [10,11]. Consequently, an HA-only model may omit evolutionary and functional information carried by NA. Antigenic behavior also depends on the structural context, residue interactions, glycosylation-associated changes, and the expansion or decline of viral clades.

Previous computational studies have used phylogenetic fitness models, mutation-based regression, deep sequence models, protein language models, and combined antigenicity and dominance predictions [12,13,14,15,16,17,18]. These approaches established the value of sequence surveillance, antigenicity predictions, and evolutionary forecasting. Nevertheless, most available methods address only part of the vaccine-selection problem. They commonly emphasize HA, treat antigenicity and future dominance as separate tasks, omit the explicit residue-contact structure, or provide limited uncertainty information for candidate ranking. Generative approaches can also explore the possible future sequence variation, but their outputs must be constrained and used at an aggregate level for responsible computational assessments [19,20].

This study presents FluEvoFormer, a structure-guided generative foundation model for prospective influenza antigenic evolution forecasting and vaccine strain ranking. The framework jointly encodes HA and NA sequences, incorporates residue-contact graphs, models changes in future viral dominance, predicts the vaccine–virus antigenic match, and quantifies the prediction uncertainty. A controlled discrete diffusion module produces future-like variant distributions for aggregate computational stress testing, while a clade-balanced and uncertainty-adjusted coverage score ranks eligible vaccine candidates. The rolling target-season evaluation is restricted to the influenza A(H1N1)pdm09 virus and influenza A(H3N2) virus. The broader historical corpus for the influenza A(H1N1) virus also contains pre-2009 seasonal records, which were distinguished from the pandemic-derived lineage during preprocessing.

The model was assessed through a cutoff-restricted rolling retrospective evaluation. For each target season, only information available before the vaccine-selection cutoff was used for training and candidate ranking. Held-out HI prediction and future viral dominance forecasting were evaluated first. The vaccine candidate ranking was then assessed using the empirical normalized coverage and uncertainty calibration. Component ablation and residue-level attribution were used to examine the contribution and biological interpretation of the model components. Statistical testing, the external clinical correlation, and the computational efficiency were also evaluated. This design assesses FluEvoFormer as a candidate prioritization and decision-support framework for subsequent laboratory and public health reviews.

The principal contributions are as follows:

1. A joint HA–NA sequence and structure representation integrates complementary evolutionary information from both influenza surface proteins.
2. A unified forecasting framework combines future dominance predictions, vaccine–virus antigenicity estimation, and pair-specific uncertainty.
3. A controlled future-like variant simulator and clade-balanced coverage score assess the candidate robustness under plausible antigenic drift scenarios without reporting generated viral sequences.
4. A cutoff-restricted rolling evaluation examines the antigenicity, dominance, vaccine ranking, calibration, interpretability, clinical association, and computational efficiency across target seasons involving the influenza A(H1N1)pdm09 virus and influenza A(H3N2) virus.

**Figure 1:**
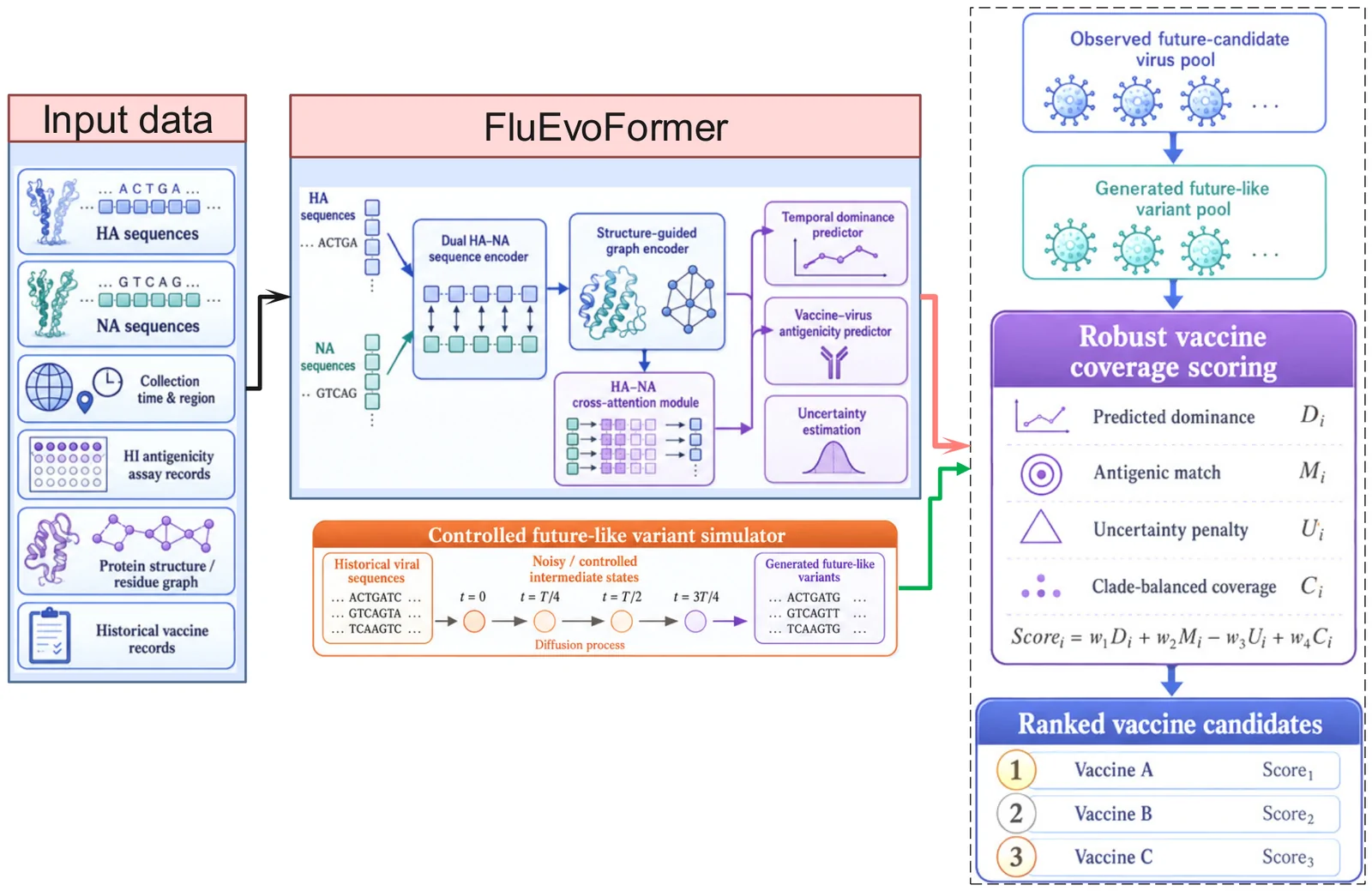
Conceptual workflow of the proposed FluEvoFormer framework. The **left column** shows the biological and surveillance inputs: the HA and NA sequences, collection time and region, HI measurements, protein structures or residue-contact graphs, and historical vaccine records. The central panel summarizes dual HA–NA sequence encoding, structure-guided graph learning, temporal dominance forecasting, vaccine–virus antigenicity predictions, uncertainty estimation, and controlled future-like variant stress testing. The **right column** shows how the observed and future-like virus pools are combined through an uncertainty-adjusted and clade-balanced coverage score to rank vaccine candidates. The lower panels indicate the intended outputs: antigenic-evolution forecasting, vaccine candidate prioritization, and residue-level interpretation. HI, hemagglutination inhibition.

Figure 1 presents the conceptual workflow and the relationship among the biological inputs, model components, and vaccine-ranking output.

## 2. Materials and Methods

### 2.1. Study Design and Data Sources

This study evaluated FluEvoFormer as a computational framework for prioritizing seasonal influenza vaccine candidates. The source corpus for the influenza A(H1N1) virus covered 2003–2023 and therefore included both pre-2009 seasonal viruses and the pandemic-derived influenza A(H1N1)pdm09 virus lineage. These groups were distinguished during preprocessing using the collection date and the available lineage or clade annotations.

Sequence pretraining and HI-pair learning used the available historical influenza A(H1N1) virus corpus. In contrast, the rolling target-season evaluation covered 2012–2013 through 2021–2022 and therefore assessed influenza A(H1N1)pdm09 virus circulation. The H1N1 dominance and vaccine-ranking results reported for those seasons consequently refer to the influenza A(H1N1)pdm09 virus. Records for the influenza A(H3N2) virus were analyzed separately throughout.

The influenza B virus was not included because the investigation was predefined around influenza A virus subtype-specific HA–NA pairing, antigenicity labels, candidate eligibility rules, and rolling evaluation seasons. Although the influenza B virus generally evolves antigenically more slowly than the influenza A(H3N2) virus, it comprises antigenically and evolutionarily distinct B/Victoria and B/Yamagata lineages that require lineage-specific sequence, HI, vaccine-composition, and prevalence datasets [21,22]. A valid influenza B virus extension would therefore require separate model training, candidate construction, and retrospective validation rather than the direct application of an influenza A virus evaluation. Only information available before each vaccine-selection cutoff was used for model training and candidate ranking.

Five data groups were included: influenza sequence records, antigenicity measurements, historical vaccine composition records, vaccine-effectiveness and disease-burden indicators, and protein structure resources. The HA and NA sequences and associated metadata were obtained from GISAID and the NCBI Influenza Virus Database [23,24]. HI measurements were obtained from WHO Collaborating Centre reports, including annual and interim reports from the Worldwide Influenza Centre at the Francis Crick Institute [18,25,26]. Historical vaccine components were obtained from WHO/GISAID vaccine-composition resources [18,27]. Vaccine-effectiveness and disease-burden indicators were obtained from CDC sources and published CDC, I-MOVE, and SPSN estimates [18,28,29]. Experimental and predicted protein structures were obtained from the Protein Data Bank and AlphaFold resources [30,31]. The data sources and their roles are summarized in Table 1.

The study was conducted entirely in silico using previously collected records and published measurements. No live influenza viruses, infectious clinical material, cell cultures, reverse-genetics constructs, viral propagation experiments, animal experiments, or field specimen collections were performed. Laboratory containment procedures and experimental personal protective equipment were therefore not applicable to the computational analyses reported here.

### 2.2. Data Processing and Biological Labels

The HA and NA sequences were filtered to remove non-human records, uncertain subtype annotations, abnormal sequence lengths, missing collection dates, and incomplete records. Duplicate protein sequences were collapsed within each season, while their occurrence counts were retained for estimating seasonal prevalence. The HA and NA records were paired when both proteins were available for the same isolate, and subtype-specific alignments were used to preserve biologically corresponding residue positions. For H1N1, records collected before the emergence of the 2009 pandemic lineage were retained as historical seasonal A/H1N1 observations, whereas pandemic-derived and subsequent seasonal records were assigned to A(H1N1)pdm09. Pre-2009 seasonal A/H1N1 records were used as historical training information, but were not interpreted as target-season A(H1N1)pdm09 observations. In the Results section, the general label A/H1N1 is retained for analyses performed on the combined historical corpus, while the target-season dominance and vaccine-ranking results are identified as A(H1N1)pdm09.

The vaccine and circulating-virus strain names in the HI reports were matched to sequence records using strain identifiers, subtype information, and collection metadata. Pairs without reliable sequence matches were excluded. When several HI measurements were available for the same vaccine–virus pair, their geometric mean was calculated before the log-scale transformation. Values closer to the homologous vaccine titer represented a stronger antigenic similarity [14,18,32].

The observed seasonal dominance was defined as the relative frequency of each retained sequence within the corresponding October–March influenza season. Protein structures were aligned to subtype-specific sequences, and residue-contact graphs were constructed using an 8 Å distance threshold. These graphs represented spatial neighborhoods among residues and were used as structure-guided learning inputs; they do not imply that an experimentally resolved structure was available for every isolate. Detailed preprocessing counts and graph-construction characteristics are reported in Supplementary Tables S1–S3.

### 2.3. Rolling Retrospective Evaluation

For each target winter season, only the sequence records, HI measurements, vaccine records, and associated metadata available before 1 February of the target year were used for training. Candidate strains were selected from eligible isolates observed during the preceding 36 months. The trained model then ranked candidates for the following October–March season. This design prevented information from the target season from entering model development and approximated the prospective vaccine candidate assessment [18].

Cutoff-specific sequence data were separated into training and validation subsets. HI pairs available before each cutoff were divided into training, validation, and held-out test subsets. The target-season observations were used only for the retrospective evaluation of the future dominance and vaccine coverage. The complete season-wise design, candidate windows, and evaluation labels are reported in Supplementary Table S4.

### 2.4. FluEvoFormer Framework

**Figure 2:**
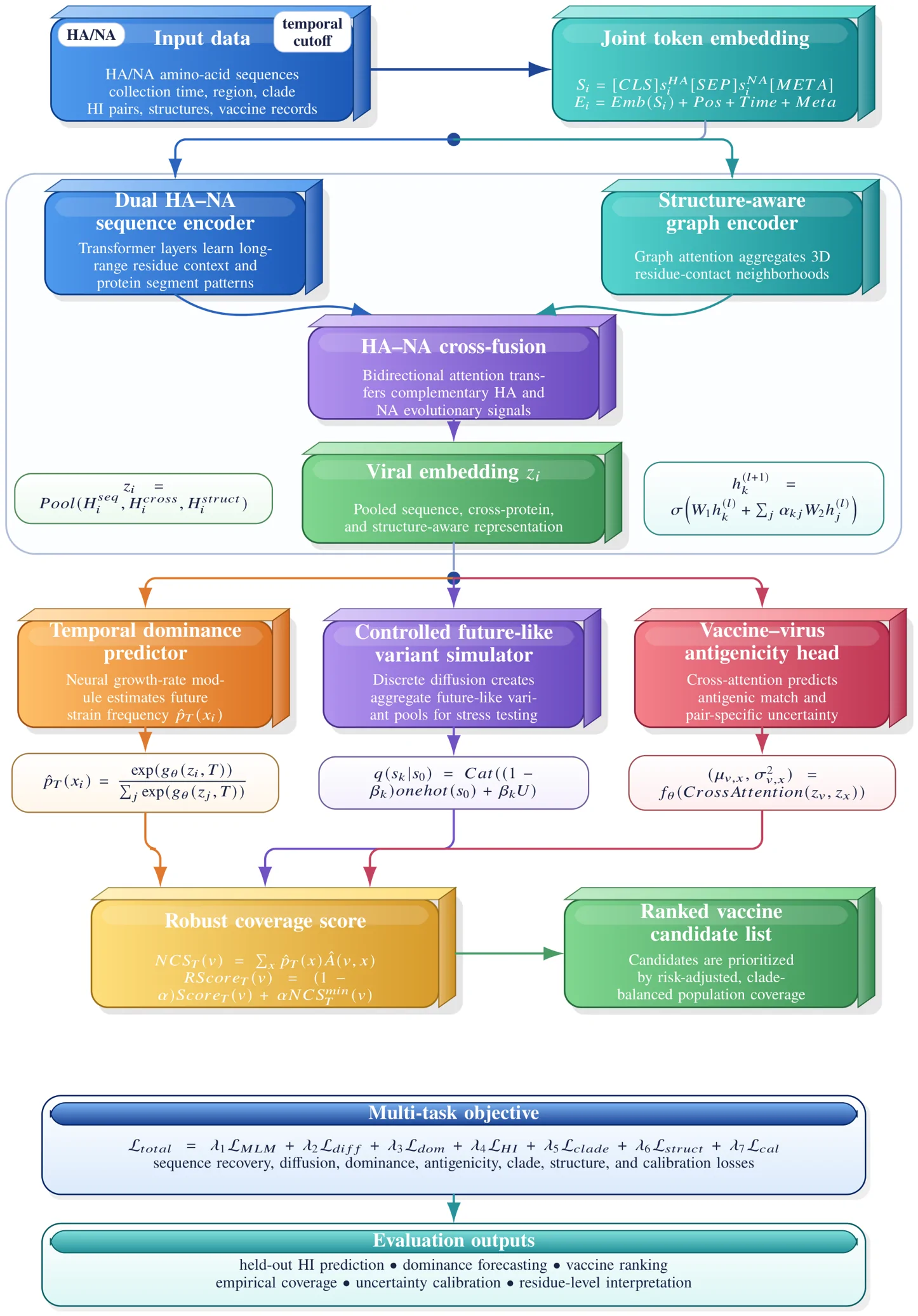
Detailed architecture of FluEvoFormer. The HA and NA sequences, temporal metadata, HI pairs, protein structures, and vaccine records are converted into a joint token representation. The dual sequence encoder and structure-aware graph encoder are fused into a common viral embedding. This embedding is processed by the temporal dominance predictor, controlled future-like variant simulator, and vaccine–virus antigenicity and uncertainty heads. Their outputs are combined in the robust coverage score to produce a ranked vaccine candidate list. The lower blocks show the multi-task training objective and the evaluated outputs.

FluEvoFormer combines sequence, structural, temporal, and antigenicity information within one vaccine-ranking framework. Its biological and computational workflow is shown in Figure 2.

The first component jointly represents the HA and NA sequences so that the model can learn both HA-centered antigenic information and complementary NA-associated evolutionary signals. The second component uses residue-contact graphs to incorporate the structural neighborhood of each amino acid rather than treating the protein only as a linear sequence. A cross-protein fusion component then combines HA and NA information into a shared viral representation.

A temporal component estimates which strains are more likely to increase in prevalence during the target season. A vaccine–virus comparison component estimates the expected antigenic match between each vaccine candidate and each circulating-virus candidate and also reports the prediction uncertainty. Finally, a controlled generative component creates aggregate future-like variant distributions for stress-testing candidate coverage under plausible drift scenarios. Generated viral sequences are not reported or treated as laboratory-ready candidates.

The complete mathematical formulation, component-level equations, loss functions, and module-specific diagrams are provided in the Supplementary Methods and Supplementary Figures S1–S3.

### 2.5. Vaccine Candidate Ranking and Evaluation

For each target season, the model estimated two principal quantities: the expected future prevalence of each circulating-virus candidate and the expected antigenic match between each vaccine candidate and that future viral population.

In this study, computational vaccine coverage denotes a model-derived estimate of how well a candidate vaccine is expected to antigenically match the predicted future circulating virus population after each virus has been weighted by its predicted dominance. It is a candidate-ranking surrogate and is not a direct measurement of the clinical protection, immune response, or vaccine effectiveness.

The predicted dominance and antigenic-match values were combined into a normalized coverage score (NCS). Candidate scores were reduced when the vaccine–virus predictions were uncertain and were additionally balanced across viral clades. This prevented a candidate from ranking highly by matching only one dominant clade while providing weak coverage of other epidemiologically relevant clades.

For a retrospective evaluation, the empirical normalized coverage was calculated from the observed target-season dominance and the measured HI-derived antigenic similarity. This retrospective score was used to determine whether the model-ranked candidate provided broader observed coverage than the historical recommendation. The complete mathematical definition of the ranking score and the training and ranking procedures are provided in the Supplementary Methods and Supplementary Algorithms S1 and S2.

The model was compared with temporal, phylogenetic, conventional machine-learning, deep-learning, protein-language-model, and simplified scoring baselines. These included last-season dominance, clade-frequency and phylogenetic ranking, random forest, XGBoost, a convolutional neural network (CNN), a long short-term memory network (LSTM), a transformer, protein language model regression, an HA-only multiple-sequence-alignment (MSA) transformer, dominance-only scoring, and antigenicity-only scoring. Detailed baseline inputs and comparison purposes are reported in Supplementary Table S5.

The antigenicity prediction was evaluated using the mean absolute error, root mean squared error, Pearson correlation, Spearman correlation, and calibration error. Future dominance was evaluated using the Kullback–Leibler divergence, root mean squared error, top-*k* recall, and rank correlation. The vaccine ranking was assessed using the empirical normalized coverage, season-wise win rate, best-tested-strain rate, and the mean rank of the selected strain. Uncertainty was evaluated using the expected calibration error (ECE), 95% prediction-interval coverage, and negative log-likelihood (NLL).

### 2.6. Model Training, Implementation, and Statistical Analysis

Training was conducted in four stages. First, the HA–NA sequence encoder was pretrained using the masked amino acid recovery. Second, the vaccine–virus predictor was trained using cutoff-restricted HI pairs. Third, the temporal component was trained using historical seasonal sequence frequencies. Fourth, all components were jointly optimized using the sequence, structure, dominance, antigenicity, clade, generative, and calibration objectives. The validation loss was used for checkpoint selection, and target-season test data remained unseen during training.

The models were implemented in Python (3.14.6) using PyTorch (2.13), scikit-learn (1.9.0), and XGBoost (3.3.0) [33,34,35]. Neural models were optimized with AdamW [36]. Training was performed on one NVIDIA A100 GPU with a 40 GB memory. The detailed architecture settings, optimization parameters, training duration, and implementation values are reported in Supplementary Table S6.

Paired season-wise comparisons were performed using Wilcoxon signed-rank tests. The Benjamini–Hochberg procedure was used to control the false discovery rate across multiple comparisons [37,38]. The Pearson correlation was used for approximately linear associations, and the Spearman correlation was used for monotonic associations when distributional assumptions were less appropriate [39]. All of the statistical tests and correlations used the cutoff-restricted retrospective evaluation outputs.

## 3. Results

### 3.1. Dataset Characterization

Table 2 summarizes the final analytical dataset used for training, validation, and the rolling retrospective evaluation. The historical A/H1N1 source corpus contained both pre-2009 seasonal A/H1N1 records and A(H1N1)pdm09 records because the source period extended from 2003 to 2023. It contained 23,736 unique HA sequences and 18,920 matched NA sequences. The A/H3N2 corpus contained 28,546 unique HA sequences and 21,860 matched NA sequences. The antigenicity dataset included 70,631 A/H1N1 and 63,299 A/H3N2 HA-pair HI records, of which 58,420 and 51,870 pairs, respectively, had matched HA–NA information. Antigenicity metrics labeled A/H1N1 therefore describe the available combined historical H1N1 corpus. In contrast, all dominance and vaccine-ranking outcomes for the 2012–2013 through 2021–2022 target seasons refer to A(H1N1)pdm09. In total, 93,062 sequence-mapped structure graphs were generated for residue-level graph learning. These data supported HI-based antigenicity predictions, future dominance forecasting, and retrospective vaccine candidate ranking.

**Figure 3:**
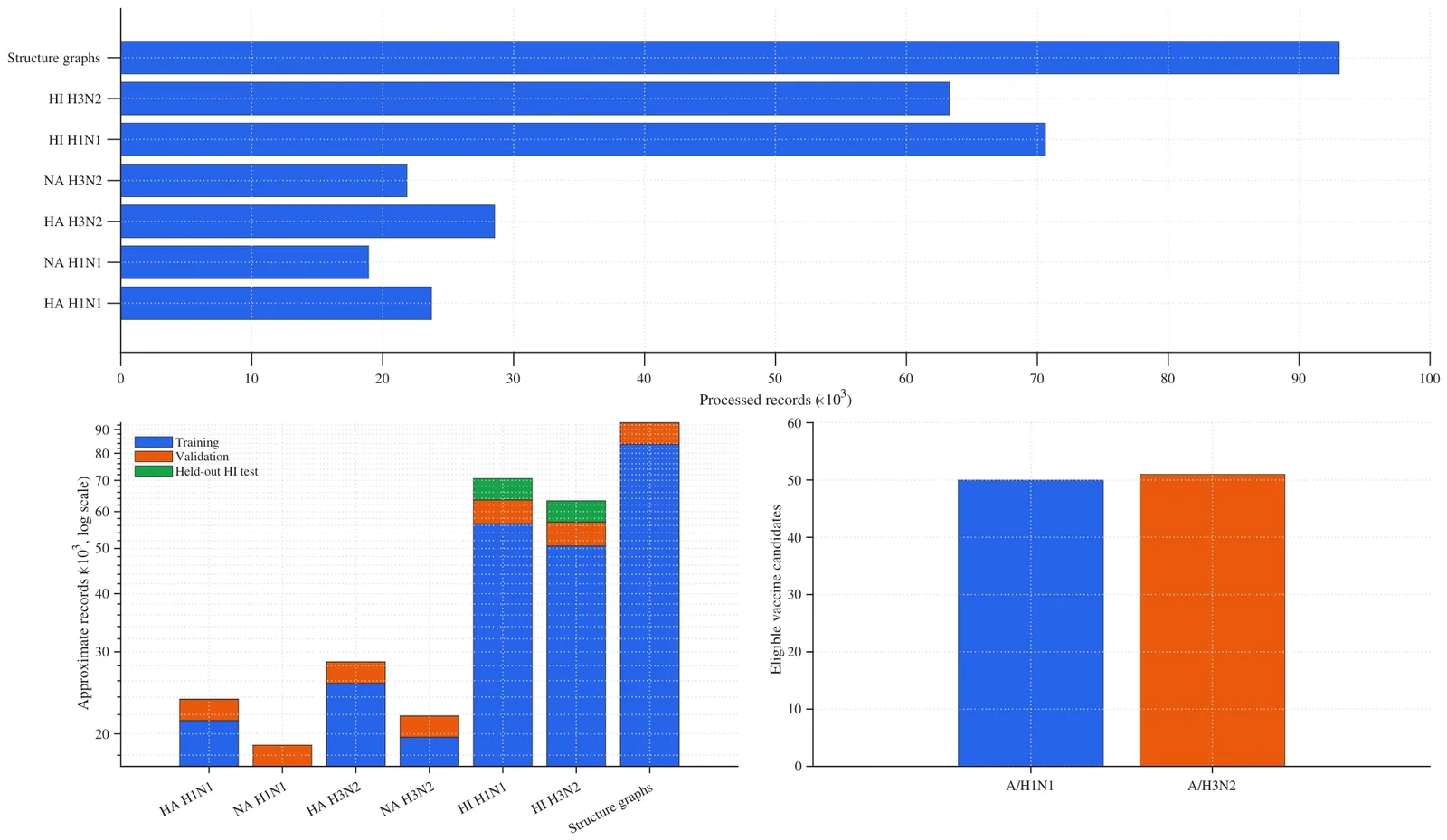
Characterization of the final cutoff-restricted analytical dataset. The upper panel reports the numbers of processed HA sequences, matched NA sequences, HI vaccine–virus pairs, and sequence-mapped structure graphs. The lower-left panel shows the approximate allocation of records to the training, validation, and held-out HI test sets on a logarithmic scale. The lower-right panel compares the numbers of eligible vaccine candidates available for the influenza A(H1N1) and A(H3N2) virus rolling evaluations. HI, hemagglutination inhibition.

Figure 3 further shows that the retained records provided broad, but uneven, coverage across sequence, antigenicity, and structure-guided learning tasks. The larger number of HA and HI records allowed for a direct comparison with HA-centered vaccine-selection baselines, while the matched NA subset enabled the proposed dual-surface protein extension. The final dataset therefore provided enough subtype-specific and season-specific coverage for cutoff-based retrospective testing.

### 3.2. Antigenicity Prediction Performance

Table 3 reports the held-out vaccine–virus antigenicity prediction results. Performance errors are reported as the mean absolute error (MAE) and root mean squared error (RMSE), for which lower values indicate a better prediction. FluEvoFormer achieved the strongest performance among the evaluated models for both subtypes. For the historical influenza A(H1N1) virus corpus, the model reduced the MAE to 0.389 and the RMSE to 0.548, with a Pearson correlation r=0.80 and a Spearman correlation ρ=0.77. For the influenza A(H3N2) virus corpus, the model achieved an MAE of 0.456 and an RMSE of 0.631, with a Pearson correlation r=0.75 and a Spearman correlation ρ=0.72. The A/H3N2 task was harder than A/H1N1, as expected, because A/H3N2 has stronger antigenic drift and more complex clade turnover.

**Figure 4:**
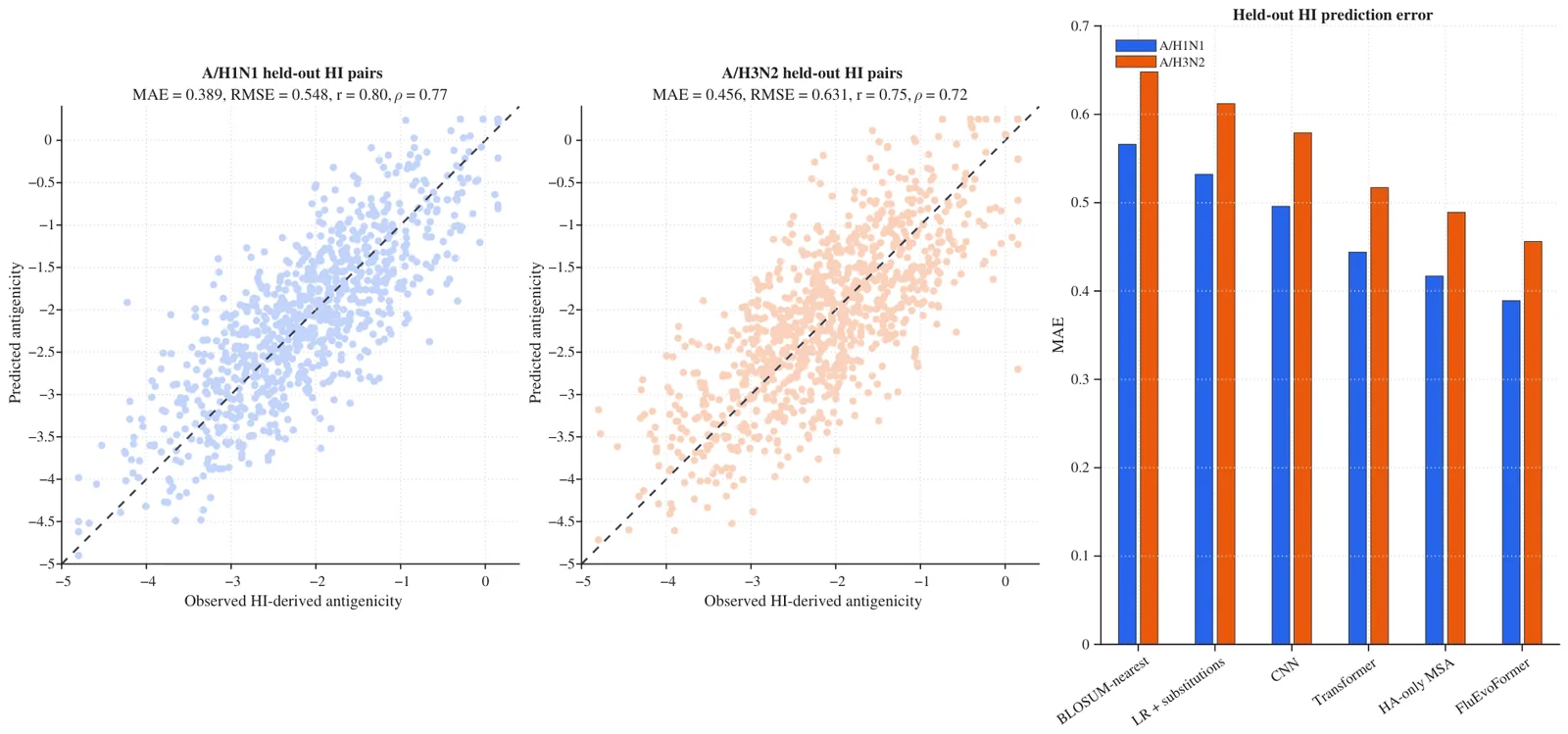
Held-out vaccine–virus antigenicity prediction results. Blue points and bars denote A/H1N1, whereas orange points and bars denote A/H3N2. The left and middle panels compare the observed and FluEvoFormer-predicted HI-derived antigenicity for the historical influenza A(H1N1) virus corpus and the influenza A(H3N2) virus corpus, respectively; the diagonal line represents the ideal agreement. The right panel compares the mean absolute error across the BLOSUM-nearest, linear-regression, CNN, transformer, HA-only MSA-transformer, and FluEvoFormer models. A lower MAE indicates a better prediction. HI, hemagglutination inhibition; MAE, mean absolute error; RMSE, root mean squared error.

Compared with the HA-only MSA-transformer baseline, FluEvoFormer reduced the MAE by 6.7% for A/H1N1 and 6.7% for A/H3N2. The gain was moderate, but consistent, which is appropriate because both methods used strong sequence-based antigenicity information and the HI labels are assay-derived and noisy. The lower calibration error of FluEvoFormer also indicates that the uncertainty-aware antigenicity head improved the reliability, not only the point prediction accuracy. Figure 4 shows that the predicted values followed the observed HI-derived antigenicity trend for both subtypes, with a broader scatter for A/H3N2.

### 3.3. Future Dominance Prediction Performance

Table 4 compares the future dominance predictions across rolling seasons. FluEvoFormer achieved the lowest KL divergence and the highest top-*k* recall for both subtypes. For A/H1N1, the model reduced the KL divergence to 0.255 and improved Recall@20 to 0.81. For A/H3N2, the model reduced the KL divergence to 0.289 and improved Recall@20 to 0.80. The improvement over the last-season dominance was larger for A/H3N2, where clade replacement and antigenic drift create stronger temporal uncertainty.

**Figure 5:**
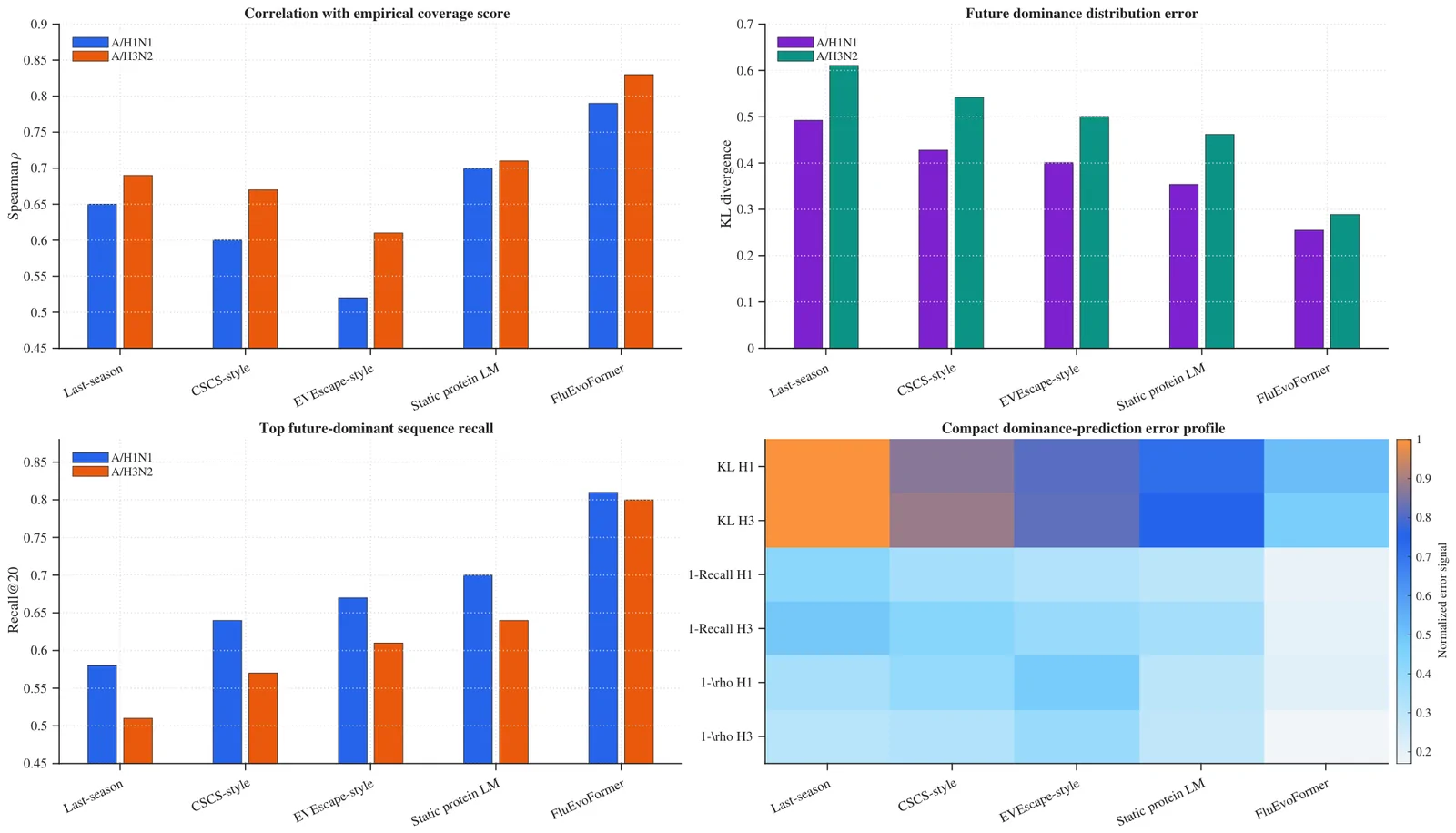
Future viral dominance predictions across the rolling target seasons. The upper-left panel shows the Spearman association between the predicted dominance and the empirical vaccine coverage. The upper-right panel compares the Kullback–Leibler divergence between the predicted and observed dominance distributions. The lower-left panel reports Recall@20 for the recovery of the most abundant future sequences. The lower-right heat map summarizes the normalized error signals based on the KL divergence, 1−Recall@20, and 1−ρ. A lower KL divergence and normalized error, and a higher Recall@20 and Spearman correlation, indicate a better performance.

The dominance results show that the static sequence scores alone did not fully capture future seasonal expansion. The proposed temporal module improved both the distributional accuracy and the top-dominant strain recovery, which is important because vaccine ranking depends more on high-frequency future viruses than on rare strains. Figure 5 supports this pattern by showing a lower dominance-distribution error, a higher Recall@20, and a stronger correlation with the empirical coverage score for FluEvoFormer.

### 3.4. Vaccine Candidate Ranking and Coverage Score

Table 5 presents the season-wise comparison between historical recommendations and FluEvoFormer-ranked candidates. Coded strain identifiers are used in the table for compact reporting. Supplementary Table S7 defines these identifiers and summarizes the reporting basis for historical recommendations and FluEvoFormer-ranked candidates. The proposed model improved the normalized empirical coverage score in seven out of 10 A(H1N1)pdm09 seasons and nine out of 10 A/H3N2 seasons. The mean gain was +0.020 for A/H1N1 and +0.043 for A/H3N2. The stronger A/H3N2 gain is consistent with the higher value of prospective modeling under rapid antigenic drift.

**Figure 6:**
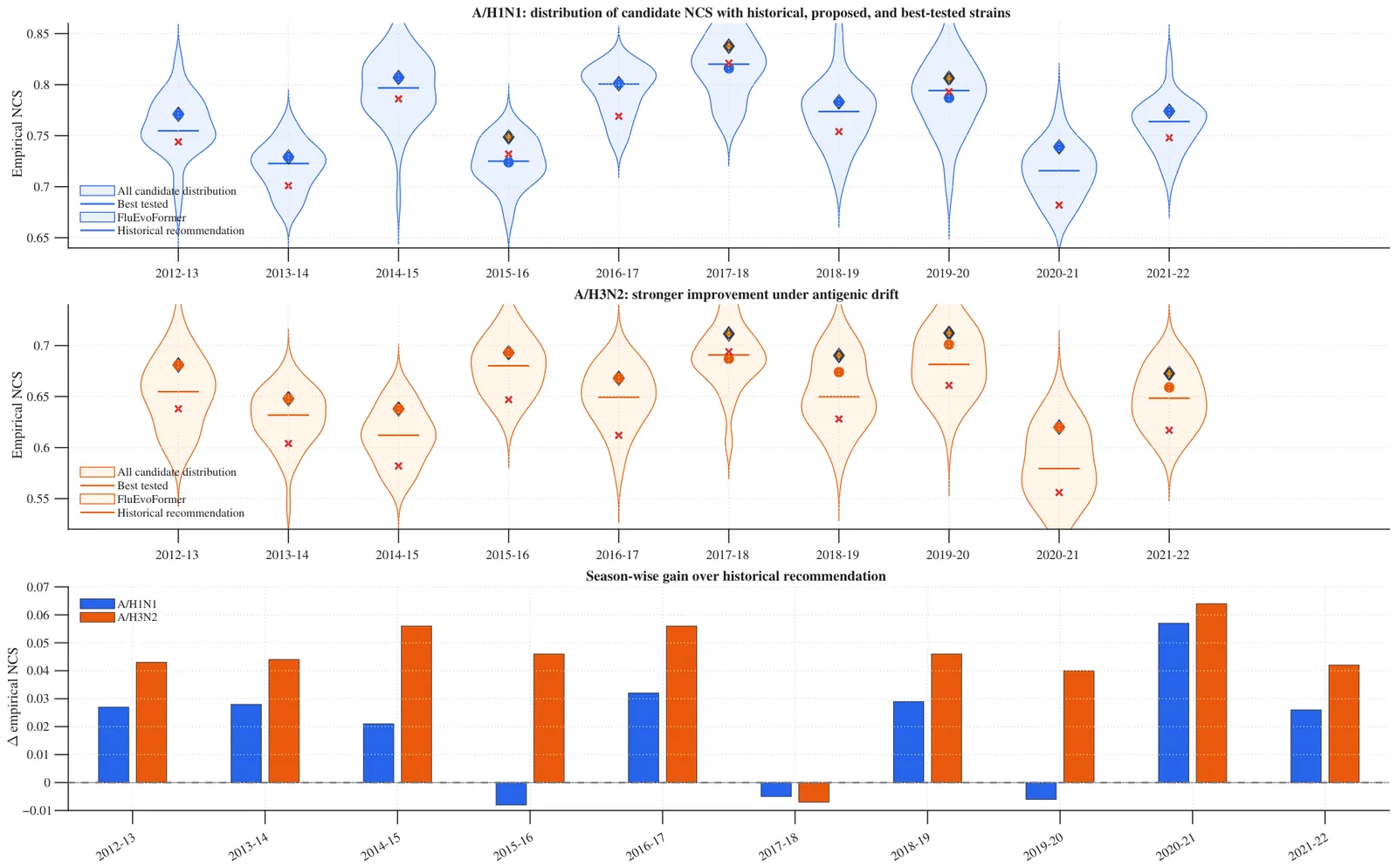
Season-wise empirical normalized coverage score distributions for vaccine candidates. The upper and middle panels show candidate-score distributions for the influenza A(H1N1)pdm09 virus and influenza A(H3N2) virus evaluations, respectively. Markers identify the historical recommendation, the FluEvoFormer-ranked candidate, and the best retrospectively tested candidate within each season. The lower panel reports the difference between the proposed and historical empirical scores; positive values indicate higher retrospective coverage for the FluEvoFormer candidate. NCS, normalized coverage score.

Figure 6 visualizes the same ranking behavior across seasons. The proposed candidates usually appear closer to the upper part of the candidate coverage distribution than the historical recommendations. The improvement was not uniform across every season, which suggests that the proposed framework is useful as a decision-support and candidate-prioritization tool rather than a replacement for experimental strain selection.

**Figure 7:**
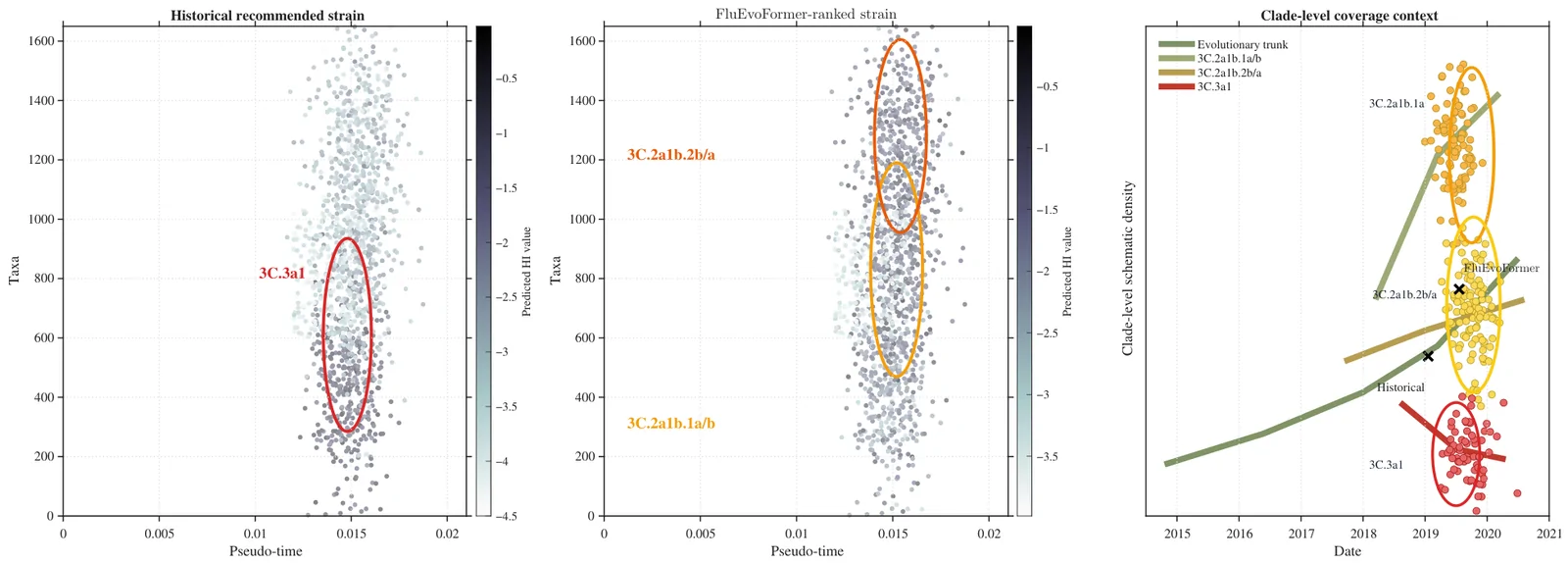
Clade-level interpretation of the 2019–2020 influenza A(H3N2) virus ranking result. The left panel shows the predicted HI-derived antigenicity profile of the historical vaccine recommendation across the circulating-virus distribution. The middle panel shows the corresponding profile for the FluEvoFormer-ranked candidate; values closer to zero indicate a stronger predicted antigenic match. The right panel places the 3C.3a1, 3C.2a1b.1a/b, and 3C.2a1b.2b/a clusters in their temporal and clade-level context. The historical candidate predominantly aligns with 3C.3a1, whereas the model-ranked candidate shows a broader predicted coverage of the 3C.2a1b clusters. Only the predicted antigenicity and aggregate clade-level patterns are shown; no generated viral sequences are reported. HI, hemagglutination inhibition.

To explain the A/H3N2 ranking behavior, the 2019–2020 season was examined as a clade-level case study. Table 5 shows that the proposed selection improved the normalized empirical coverage score from 0.661 to 0.701 for this season. Figure 7 shows that the historical recommendation mainly aligned with the 3C.3a1 cluster, whereas the FluEvoFormer-ranked strain provided broader predicted antigenic coverage across the 3C.2a1b.1a/b and 3C.2a1b.2b/a clusters. This clade-level result explains one representative A/H3N2 gain and complements the season-wise ranking results in Figure 6.

### 3.5. Overall Comparison with Baseline Vaccine Selection Strategies

Table 6 compares the overall vaccine ranking performance. FluEvoFormer obtained the highest mean empirical NCS for both subtypes. For A/H1N1, the mean empirical NCS improved from 0.733 with last-season dominance and 0.766 with direct dominance-plus-antigenicity scoring to 0.773 with the proposed model. For A/H3N2, the improvement was stronger, with the mean empirical NCS increasing from 0.600 with last-season dominance and 0.654 with direct dominance-plus-antigenicity scoring to 0.667 with FluEvoFormer.

The ranking results show that neither dominance nor antigenicity alone was sufficient. Antigenicity-only scoring improved over last-season dominance, but it ignored the future prevalence of the matched viruses. Direct dominance-plus-antigenicity scoring was stronger, but the proposed model further improved the rank quality by adding HA–NA fusion, structure-guided encoding, uncertainty adjustments, clade-balanced scoring, and generated future-like stress testing.

### 3.6. Correlation with Vaccine Effectiveness and Disease Burden

Table 7 evaluates whether the predicted normalized coverage score agrees with external vaccine-effectiveness and disease burden indicators [28,29]. The 2020–2021 season was excluded from the vaccine-effectiveness correlation because influenza circulation was unusually low during the SARS-CoV-2 pandemic, and the seasonal VE estimates were not sufficiently stable for a standard comparison [28,40]. The predicted NCS showed a strong positive correlation with the CDC vaccine effectiveness estimates, with Pearson r=0.872, Spearman ρ=0.903, and p=0.0011. Positive correlations were also observed for I-MOVE and SPSN vaccine-effectiveness estimates. Correlations with the CDC averted illness and medical-visit indicators were moderate but positive.

**Figure 8:**
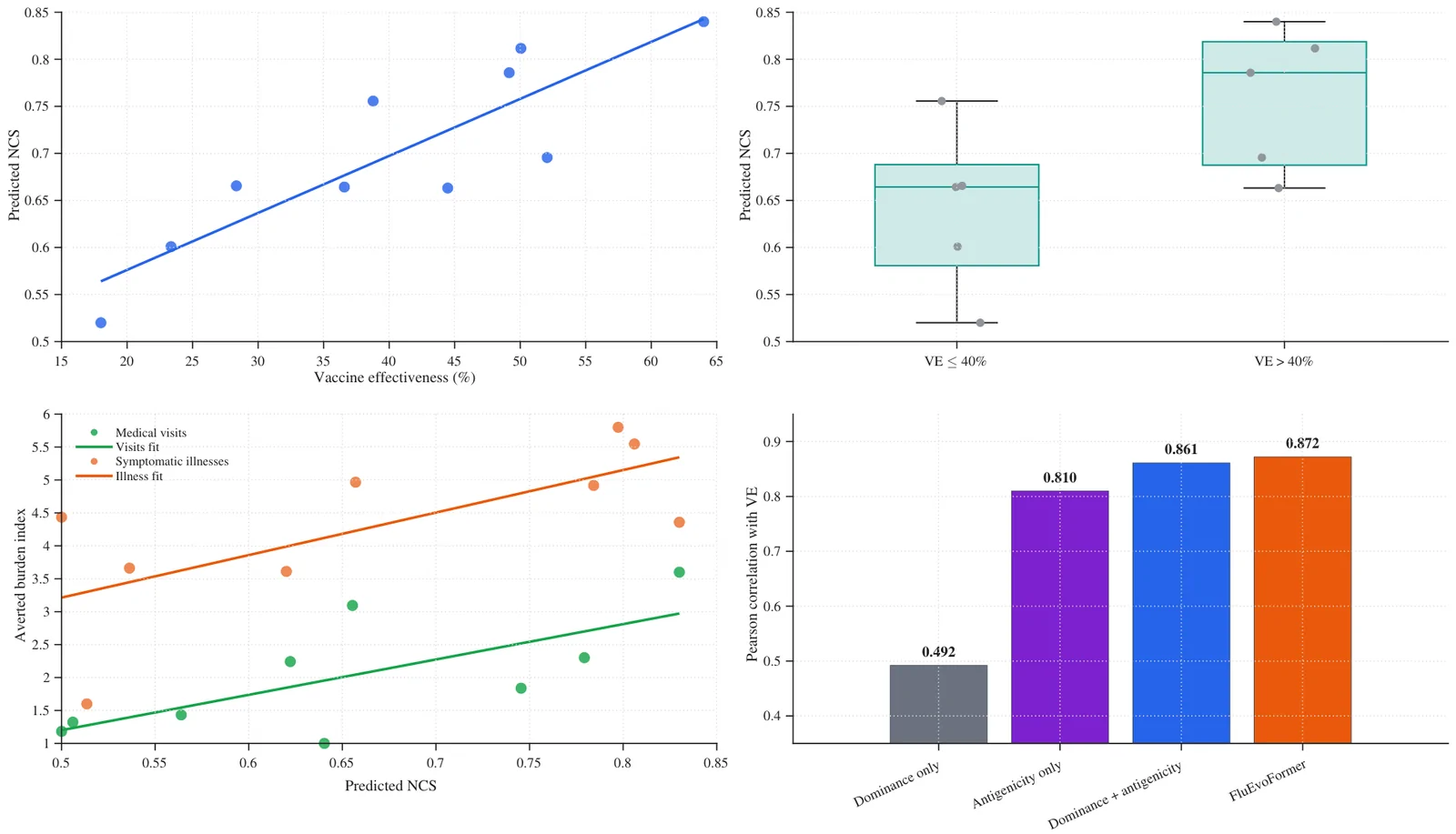
External association of the predicted normalized coverage score with vaccine-effectiveness and disease-burden indicators. The upper-left panel shows the association between the predicted NCS and CDC vaccine-effectiveness estimates. The upper-right panel compares the NCS values between seasons with a vaccine effectiveness of 40% or less and seasons with a vaccine effectiveness above 40%. The lower-left panel relates the NCS to CDC estimates of averted illnesses and medical visits. The lower-right panel compares the Pearson correlation with the vaccine effectiveness obtained by alternative scoring strategies. These associations provide external support for the ranking score, but do not establish a causal estimate of clinical vaccine effectiveness. NCS, normalized coverage score; VE, vaccine effectiveness.

These findings support the practical relevance of the proposed coverage score, but they do not prove direct clinical effectiveness. Vaccine effectiveness depends on several factors beyond antigenic match, including prior immunity, the vaccine platform, the production method, the age distribution, the timing, and the circulating subtype mixture. Therefore, the clinical correlation analysis should be interpreted as external support for the ranking score rather than as a substitute for epidemiological vaccine-effectiveness studies. Figure 8 visualizes the relationship between the predicted coverage score, vaccine effectiveness, and disease burden indicators.

### 3.7. Ablation Study

The ablation study in Table 8 evaluated the contribution of each major model component. Removing the NA encoder reduced the mean empirical NCS from 0.720 to 0.710, showing that NA provides useful complementary information. Removing the structure graph reduced the mean empirical NCS to 0.708, indicating that the residue-contact context supports antigenicity and dominance predictions. Removing the diffusion simulator caused the largest component-level drop among the individual modules, reducing Recall@20 from 0.805 to 0.753 and the mean empirical NCS to 0.703. This result suggests that generated future-like variant pools improve the stress testing of candidate coverage under plausible antigenic drift.

**Figure 9:**
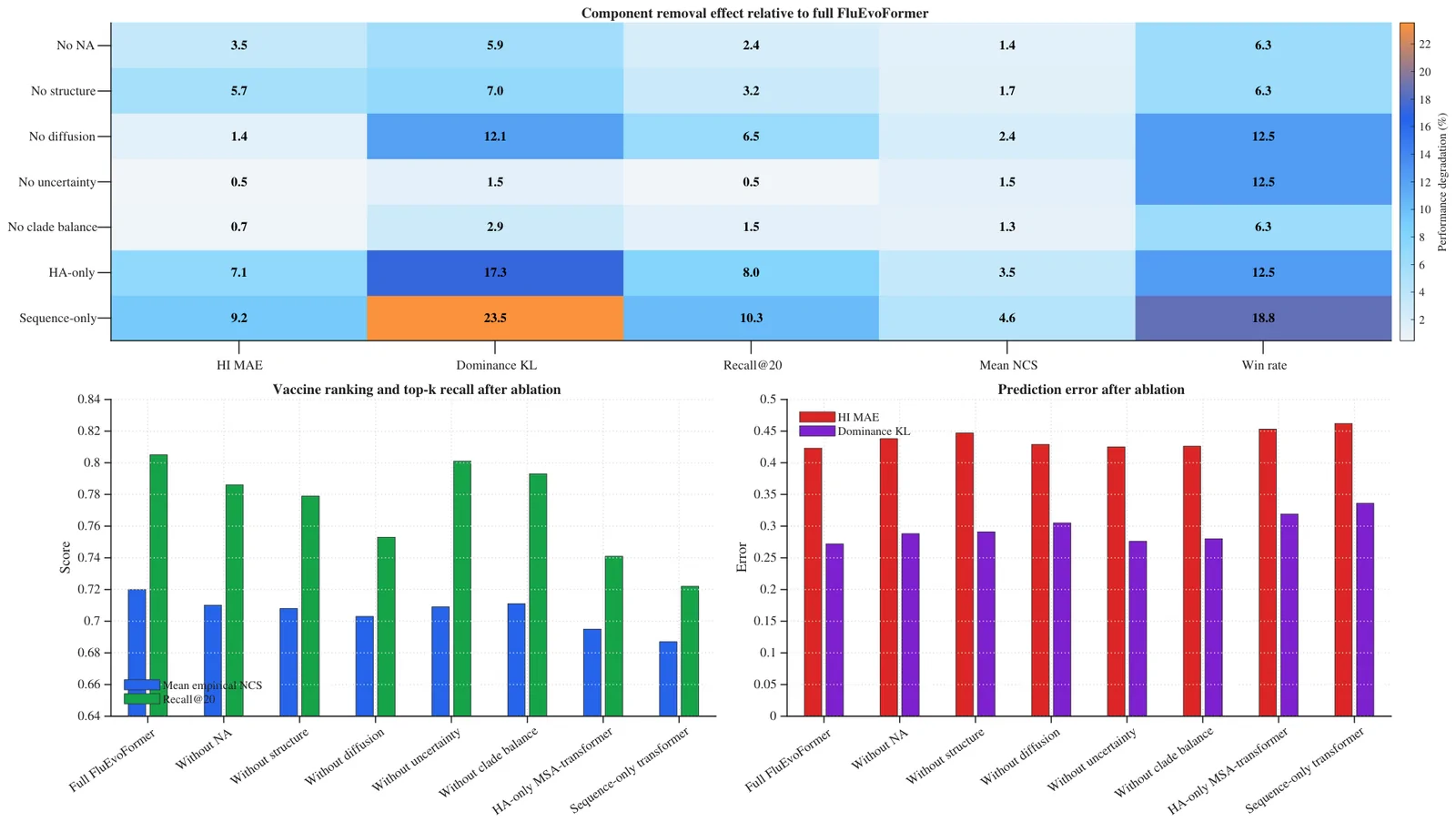
Performance changes after removal of individual FluEvoFormer components. Bold numbers in the upper heat map indicate the percentage performance degradation relative to the full model. The upper heat map summarizes changes relative to the full model in the HI MAE, dominance KL divergence, Recall@20, mean empirical NCS, and win rate. The lower-left panel compares vaccine-ranking and top-*k* recovery measures across model variants, while the lower-right panel compares antigenicity and dominance prediction errors. A lower MAE and KL divergence and a higher Recall@20, NCS, and win rate indicate a better performance. HI, hemagglutination inhibition; MAE, mean absolute error; KL, Kullback–Leibler; NCS, normalized coverage score.

The HA-only and sequence-only variants showed the largest drops. The HA-only backbone reduced the mean empirical NCS to 0.695, while the sequence-only transformer reduced it to 0.687. These results support the central design of FluEvoFormer: vaccine ranking benefits from combining the HA, NA, protein structure, temporal dynamics, uncertainty, and clade-balanced coverage instead of relying on sequence tokens alone. Figure 9 visualizes the performance drop caused by removing each major component.

### 3.8. Uncertainty and Calibration Analysis

Table 9 reports the uncertainty and calibration results. FluEvoFormer produced the lowest expected calibration error for both A/H1N1 and A/H3N2. For A/H1N1, the ECE decreased from 0.096 for the transformer baseline to 0.061 for the full model. For A/H3N2, the ECE decreased from 0.111 to 0.071. The 95% interval coverage also improved and approached the target level, reaching 0.939 for A/H1N1 and 0.934 for A/H3N2.

**Figure 10:**
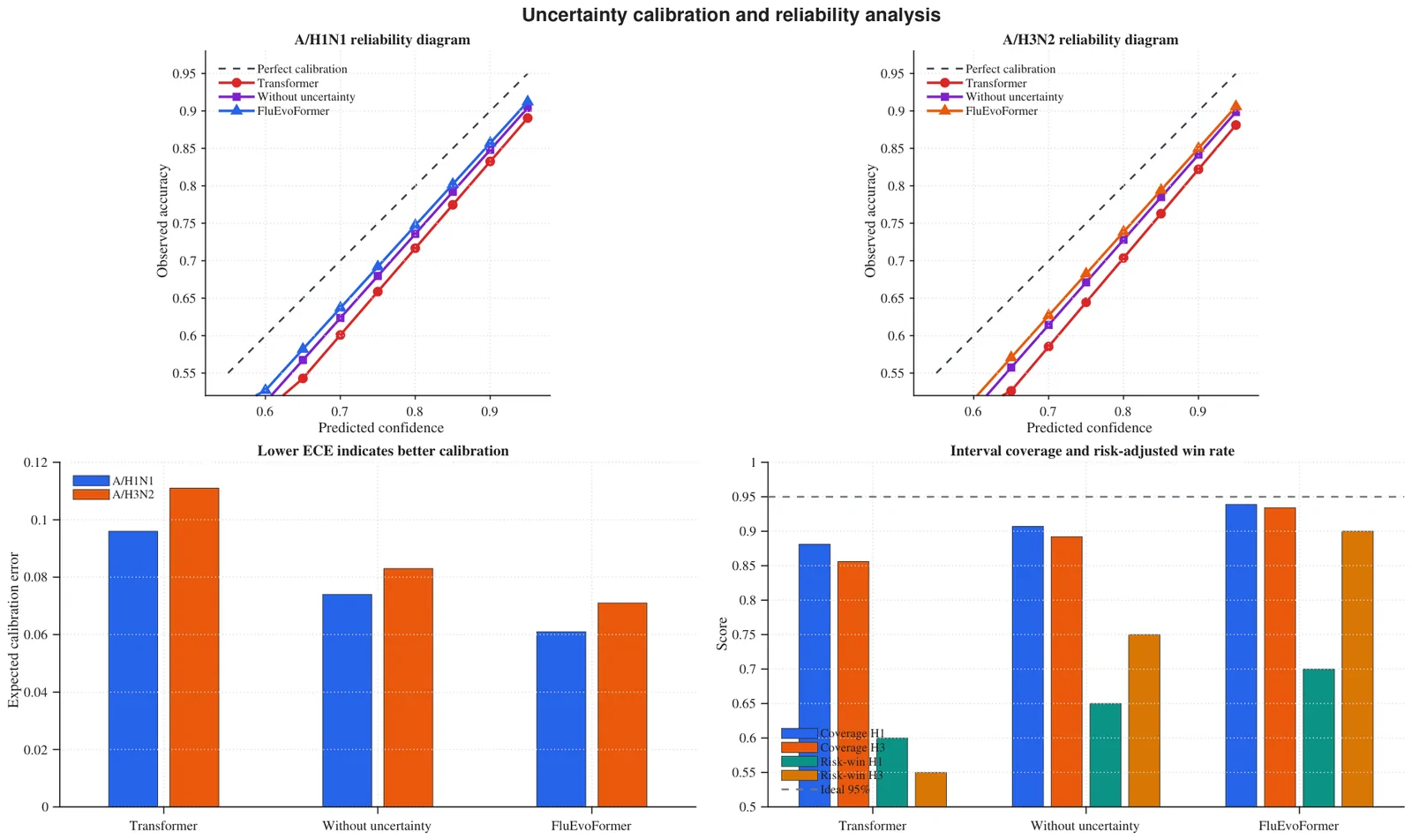
Reliability and calibration analysis of antigenicity and vaccine ranking predictions.

The risk-adjusted win rate increased when the uncertainty was included in the final score. This result is important because deterministic ranking may overvalue candidates with a high predicted antigenicity, but unstable predictions. The uncertainty penalty reduced overconfident selection and made the final recommendation more reliable under limited or noisy HI data. Figure 10 supports this conclusion through reliability diagrams and interval coverage comparisons.

### 3.9. Generated Future-like Variant Evaluation

Table 10 evaluates the generated future-like variant pools used to estimate the expected antigenic coverage and stress-test candidate rankings under model-generated future-like scenarios. The generated pools showed a moderate novelty, high clade consistency, and high plausibility-filter pass rates. A/H3N2 generated pools had a higher novelty than A/H1N1, which agrees with stronger A/H3N2 drift patterns. The effect on the predicted NCS ranged from +0.009 to +0.025, showing that generated future-like pools provided a small, but consistent, stress-testing contribution.

**Figure 11:**
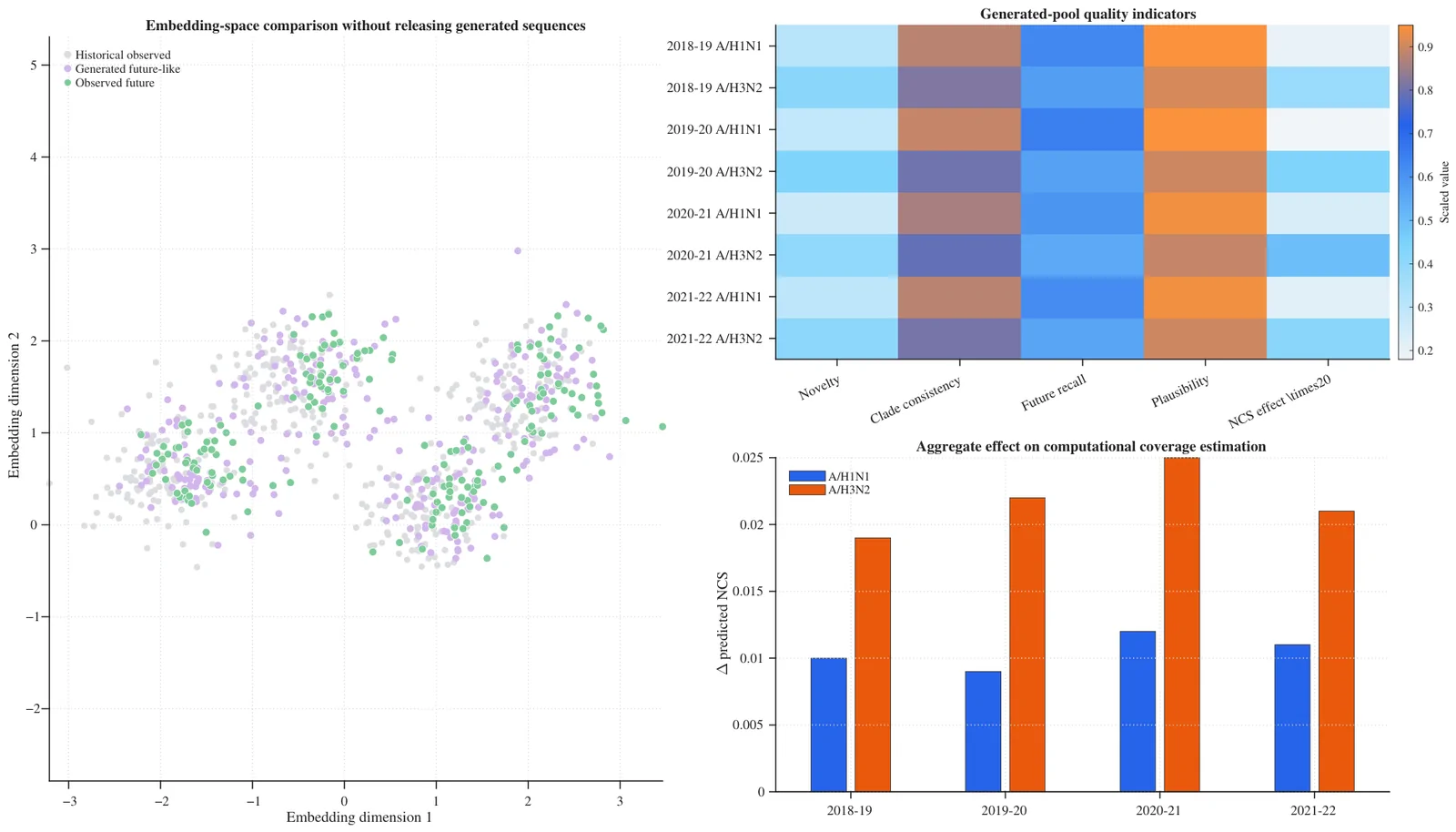
Embedding-space comparison of historical sequences, generated future-like variants, and observed future sequences.

Figure 11 compares the historical observed sequences, generated future-like variants, and observed future sequences in the embedding space. The generated pools were placed between historical and future observed distributions, which suggests that the diffusion module produced useful scenario-level variation without requiring the release of raw generated sequences. These results support the use of the generator as an aggregate computational stress-testing module, not as a source of deployable viral designs.

### 3.10. Explainability and Residue-Level Interpretation

The residue-level attribution was calculated as:

(1) $$I_{k}=\left|\frac{\partial {\hat{y}}_{v,x}}{\partial e_{k}}\right|,$$

where $I_{k}$ denotes the attribution score for residue *k*, ${\hat{y}}_{v,x}$ denotes the predicted output for vaccine candidate *v* and virus *x*, and $e_{k}$ denotes the embedding of residue *k*. Table 11 reports the most influential residue-level signals. For A/H1N1, HA-156/158 and HA-190/193 had high attribution scores and were linked with antigenic head and receptor-binding-proximal regions. For A/H3N2, HA-145/159 and HA-186/189 showed the strongest contributions, consistent with the known importance of head-domain and receptor-binding-proximal changes for antigenic variation. The NA residues showed lower, but non-negligible, attribution scores and were interpreted as secondary fitness or HA–NA balance signals.

Table 11 reports the highest model-attribution signals. Several HA signals occurred in or near the head-domain, antigenic, and receptor-binding-proximal regions previously associated with influenza antigenic variation [4,5,14]. These associations provide biological context for the model attribution, but do not establish that the identified residue clusters independently cause antigenic change. The lower NA attribution signals are interpreted as putative complementary evolutionary or HA–NA balance signals and require experimental validation.

**Figure 12:**
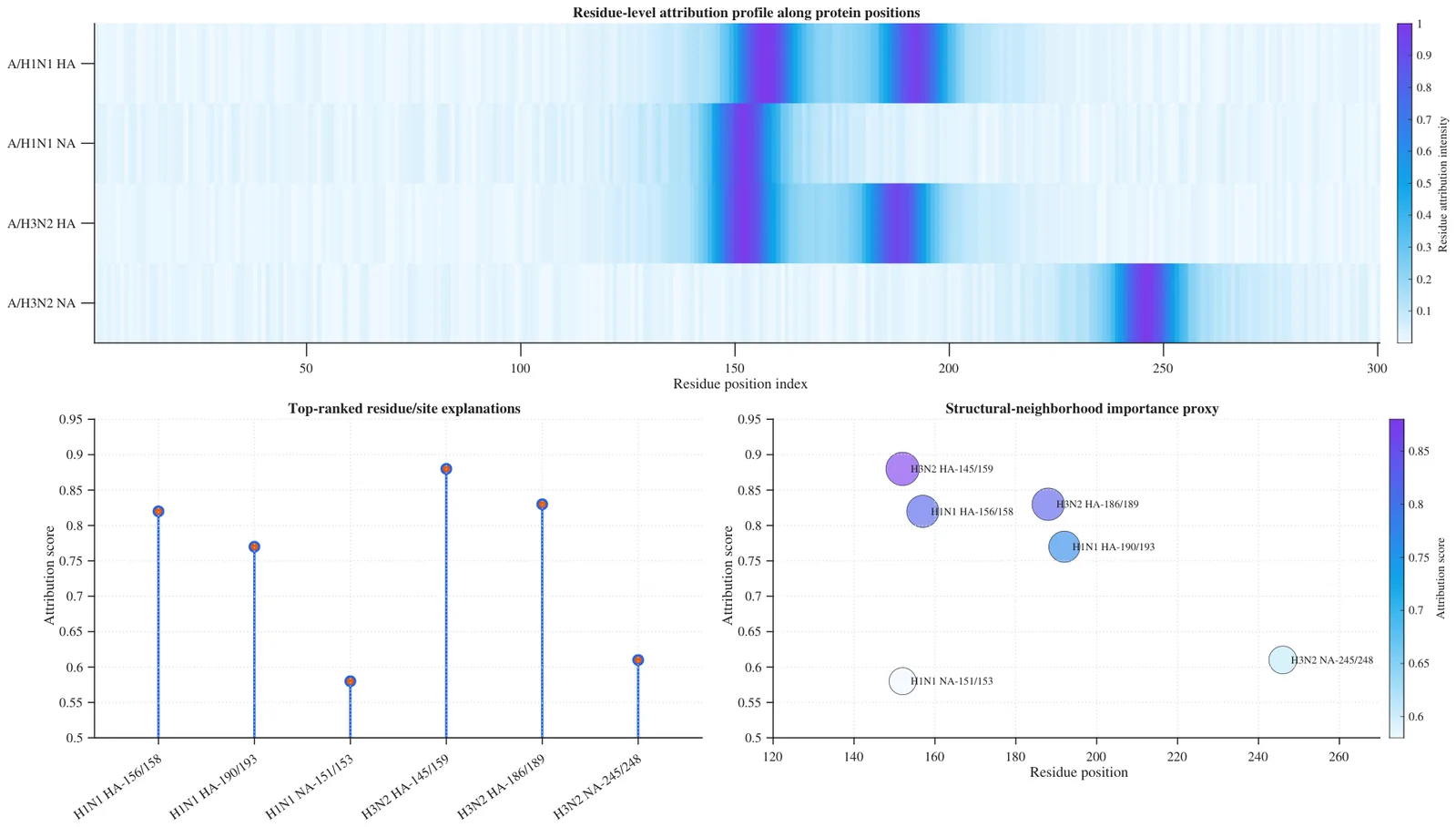
Residue-level attribution patterns for FluEvoFormer predictions. The upper heat map shows the attribution intensity across the aligned HA and NA residue positions for the influenza A(H1N1) and A(H3N2) virus datasets. The lower-left panel reports the highest-ranked residue or residue-cluster signals. The lower-right panel relates the attribution magnitude to the residue position and structural-neighborhood importance. The highlighted HA sites are concentrated near antigenic-head and receptor-binding-proximal regions, whereas the NA sites provide weaker complementary fitness and HA–NA balance signals.

Figure 12 visualizes these residue-level attribution patterns and their structural-neighborhood context.

### 3.11. Statistical Significance Analysis

Table 12 reports the paired statistical comparisons across seasons. FluEvoFormer significantly improved the empirical NCS over the historical recommendations for A/H1N1 (p=0.046), A/H3N2 (p=0.009), and the combined subtype setting (p=0.004). The gain over antigenicity-only scoring was significant for A/H3N2 and marginal for A/H1N1, suggesting that the added value of future dominance and robust scoring is strongest when antigenic drift is more pronounced. FluEvoFormer also significantly reduced the HI MAE compared with the HA-only MSA-transformer baseline and reduced the dominance KL compared with the static protein language model.

### 3.12. Computational Efficiency

Table 13 reports the computational efficiency. FluEvoFormer required 54.8 h of training and 38.6 GB of GPU memory, which was higher than the simpler baselines. However, inference required only 8.7 min per season and screened 5800 vaccine–virus pairs per minute. This computational cost remains feasible for retrospective seasonal screening and candidate prioritization because strain selection is not a real-time task.

Supplementary Figure S4 visualizes the deployment trade-off across the training time, seasonal inference time, GPU memory, and vaccine–virus pair screening throughput. The proposed model is more computationally demanding than the baselines, but the additional cost is mainly during training. Seasonal inference remains practical for candidate screening.

## 4. Discussion

The results show that FluEvoFormer improves three connected parts of prospective influenza vaccine strain selection: antigenicity predictions, future dominance forecasting, and vaccine candidate ranking. The model achieved the best held-out HI prediction performance for both A/H1N1 and A/H3N2, reduced future dominance distribution errors, and selected vaccine candidates with higher normalized empirical coverage scores than the historical recommendations in most rolling seasons. The strongest gains appeared for A/H3N2, which is biologically plausible because A/H3N2 usually shows stronger antigenic drift and more frequent clade replacement than A/H1N1 [4,15,18].

The antigenicity results indicate that paired vaccine–virus cross-attention can learn meaningful HI-derived similarity patterns from sequence- and structure-aware representations. The improvement over the HA-only MSA-transformer baseline was moderate, not exaggerated, which is expected because the baseline is already strong and the HI labels are noisy. The lower calibration error of FluEvoFormer is important because vaccine strain ranking should not depend only on point estimates. A model that reports uncertainty can penalize unreliable candidate-virus comparisons and reduce overconfident recommendations.

The dominance results support the value of temporal learning. Last-season dominance and static protein language model scores captured part of the future distribution, but they did not fully model seasonal expansion. FluEvoFormer improved the KL divergence, Recall@10, Recall@20, and Spearman correlation by learning a time-dependent dominance function from the historical sequence frequency patterns. This result agrees with the principle that vaccine selection depends not only on antigenic similarity, but also on which viral strains are likely to circulate in the target season [14,18].

The vaccine ranking results show that combining antigenicity and future dominance is more informative than using either signal alone. Antigenicity-only scoring can select a vaccine that matches rare strains, while dominance-only scoring can select a strain that tracks prevalence, but does not match antigenically. The direct dominance-plus-antigenicity score improved both subtypes, but FluEvoFormer further improved candidate ranking through HA–NA fusion, structure-guided encoding, uncertainty adjustments, generated future-like stress testing, and clade-balanced scoring. The 2019–2020 A/H3N2 case study provides a clear example. The model-selected strain achieved a broader predicted coverage across the 3C.2a1b.1a/b and 3C.2a1b.2b/a clusters, while the historical recommendation mainly aligned with the 3C.3a1 cluster. This result explains why the proposed model improved the A/H3N2 normalized empirical coverage score in that season.

The HA–NA design is one of the main reasons for the observed gain. HA remains the primary target for HI-based antigenicity, but NA contributes to viral release, fitness, immune protection, and the HA–NA functional balance [9,11,41]. The ablation study showed that removing the NA encoder reduced the NCS and dominance performance. This does not mean that NA directly determines HI antigenicity. Instead, NA appears to provide complementary evolutionary and fitness-related information that improves candidate ranking when combined with HA.

Structure-guided learning also improved the performance. Removing the residue-contact graph increased the HI error and reduced the empirical NCS. This suggests that antigenic behavior is affected not only by isolated amino acid substitutions, but also by structural neighborhoods. Residue-level attribution further supported this interpretation. Important HA residues appeared near antigenic head and receptor-binding proximal regions, while NA residues contributed weaker secondary signals linked with dominance and the HA–NA balance. These explanations make the model more interpretable and reduce the risk of treating the AI prediction as a black-box score.

The controlled future-like variant simulator added a small, but consistent, improvement. Its main value is not direct vaccine design, but aggregate stress testing. Generated future-like pools helped estimate how candidate vaccine coverage might change under plausible antigenic drift. The embedding-space analysis showed that generated variants occupied regions between historical and future observed sequences, while plausibility filters removed unstable or inconsistent outputs. The study deliberately reports only aggregate statistics and embedding-level patterns, not raw generated viral sequences. This reporting choice supports computational safety while preserving the scientific value of generative modeling.

The clinical correlation analysis provides external support for the normalized coverage score. The predicted NCS correlated positively with the CDC, I-MOVE, and SPSN vaccine-effectiveness estimates and with the CDC disease burden indicators. This suggests that antigenic coverage is a useful computational marker of seasonal vaccine performance. However, the correlation should not be interpreted as direct clinical proof. Vaccine effectiveness also depends on the prior immunity, age distribution, vaccine uptake, vaccine platform, manufacturing process, infection history, and seasonal epidemiology [1,2,28]. The 2020–2021 season was excluded from the VE correlation because influenza circulation was unusually low during the COVID-19 pandemic, which makes standard VE interpretation unreliable [28,40].

The statistical tests support the main finding. The proposed model significantly improved the empirical NCS over historical recommendations for A/H1N1, A/H3N2, and the combined setting. The improvement was stronger for A/H3N2, which is consistent with the higher burden of antigenic drift in this subtype. The A/H1N1 improvement over antigenicity-only scoring was marginal, suggesting that the added modules provide smaller, but still useful, gains when seasonal evolution is less complex. The ablation results further show that no single component explains the full improvement. The final performance came from the combined effect of dual-protein encoding, structural information, temporal dominance learning, uncertainty, clade balancing, and controlled generative stress testing.

Influenza B was not evaluated in the present study. This exclusion should not be interpreted as evidence that influenza B is evolutionarily static or unimportant for seasonal vaccine composition. Previous antigenic and molecular analyses indicate that influenza B generally evolves more slowly than A/H3N2; however, B/Victoria and B/Yamagata are antigenically distinct lineages with different patterns of transmission, antigenic drift, lineage turnover, and genome-segment reassortment [21,22]. Consequently, the lower average evolutionary rate of influenza B does not justify transferring parameters or performance estimates directly from the influenza A models. The extension of FluEvoFormer to influenza B would require lineage-specific HA–NA representation learning, separate temporal dominance labels, appropriate HI-pair construction, influenza B vaccine-candidate definitions, and an independent rolling retrospective evaluation. Influenza B was therefore excluded to preserve a coherent influenza A evaluation rather than because its antigenic evolution is negligible.

The computational efficiency analysis showed that the proposed model is more computationally demanding than the CNN, transformer, and HA-only MSA-transformer baselines. This cost is expected because the model performs multi-task learning with sequence, structure, temporal, uncertainty, and generative modules. However, seasonal inference took less than 10 min and screened thousands of vaccine–virus pairs per minute. Therefore, the model remains practical for retrospective seasonal analyses and candidate prioritization. The training cost remains feasible because vaccine strain selection is a periodic process rather than a real-time prediction task.

Overall, the results support FluEvoFormer as a computational decision-support framework for influenza vaccine strain prioritization. The model should not replace laboratory antigenic characterization, ferret antisera testing, vaccine production assessments, or public health expert reviews. Instead, it can narrow the candidate space, highlight strains with a stronger predicted future coverage, identify the clade-level mismatch risk, and provide interpretable residue-level evidence for further evaluation. The most important practical value is the ability to combine future dominance, antigenic match, uncertainty, and clade coverage into one prospective ranking score.

## 5. Limitations, Biosafety, and Ethical Considerations

This study has several limitations. The historical H1N1 training corpus includes both pre-2009 seasonal A/H1N1 and A(H1N1)pdm09 records, whereas the rolling target-season dominance and vaccine-ranking evaluation concerns A(H1N1)pdm09 and A/H3N2. The findings should therefore not be generalized to pre-2009-like seasonal H1N1 lineages considered independently.

Influenza B was also outside the predefined scope of the study. Its exclusion was not based on an assumption that influenza B lacks clinically relevant antigenic variation. Although B/Victoria and B/Yamagata have generally shown slower average antigenic drift than A/H3N2, they remain antigenically distinct and exhibit lineage-specific evolutionary and epidemiological dynamics [21,22]. The present influenza A model, candidate definitions, and performance estimates should therefore not be applied directly to influenza B. A separate influenza B analysis would require lineage-specific data preparation, model training, vaccine-candidate construction, and an independent evaluation.

First, influenza sequence repositories can contain sampling bias across countries, seasons, laboratories, and surveillance intensities. Regions with a stronger sequencing infrastructure may contribute more records than regions with lower surveillance coverage, which can affect the estimated strain dominance. The passage history, the collection timing, and incomplete metadata can also influence sequence interpretation. The cutoff-based evaluation reduces temporal leakage, but it cannot fully remove surveillance bias from historical datasets [23,24].

Second, HI assay data provide an important experimental measure of antigenic similarity, but they do not fully represent the complete human immune response. HI measurements can vary across laboratories, antisera panels, virus propagation conditions, and assay protocols. The model therefore treats HI-derived antigenicity as a useful, but incomplete, surrogate for a vaccine–virus match. The clinical correlation analysis further supports the relevance of the normalized coverage score, but vaccine effectiveness also depends on the prior immunity, age, vaccine platform, manufacturing process, vaccination timing, dose, and seasonal epidemiology [2,14,28,32].

Third, the structure-guided module uses representative experimental templates and sequence-mapped residue-contact graphs. This design improves residue-context learning, but it does not provide a fully resolved experimental structure for every isolate. Structural mapping may miss strain-specific conformational changes, glycosylation effects, and assay-dependent antigenic behavior. The residue-level explanations should therefore be interpreted as model-supported biological signals, not as direct experimental proof of causal antigenic mechanisms.

Fourth, the generated future-like variant module is used only for aggregate computational coverage estimation and stress testing. The study does not report generated viral sequences, laboratory-ready designs, or laboratory protocols. Generated variants are summarized through embedding-level distributions, clade-consistency scores, plausibility-filter rates, and predicted coverage effects. This restriction aligns the model with responsible life-science and dual-use risk-management principles, where computational outputs that could increase the misuse risk should be limited, reviewed, and reported at a safe level of detail [42,43].

Fifth, the study provides a retrospective computational evaluation, not a prospective clinical trial or laboratory vaccine-selection recommendation. The proposed model can prioritize candidate strains, identify the possible mismatch risk, and provide interpretable evidence for further review, but it cannot replace WHO strain-selection processes, laboratory antigenic characterization, ferret antisera testing, vaccine production assessments, or public health expert judgment. Prospective use would require independent validation on future seasons and multi-laboratory antigenicity testing. Any future wet-laboratory validation would require a separate institutional biosafety and biosecurity risk assessment, with containment practices and personal protective equipment selected according to the viral strain and experimental procedure. Operational use would also require integration with existing public health surveillance workflows.

From an ethical perspective, the framework is designed as a decision-support system. The model reports ranked candidates, aggregate coverage scores, uncertainty estimates, clade-level mismatch indicators, and residue-level explanations. It does not provide operational instructions for creating or modifying infectious viruses. Any public release of code, processed data, or model outputs should remove restricted metadata, avoid releasing generated sequence content, follow the access terms of the original databases, and include documentation that limits the use to surveillance, vaccine-prioritization research, and public health assessments.

## 6. Conclusions

This study presented FluEvoFormer, a structure-guided generative foundation model for prospective influenza antigenic evolution forecasting and vaccine strain selection. The framework integrated HA and NA protein sequence learning, residue-contact graph encoding, temporal dominance predictions, vaccine–virus antigenicity predictions, controlled future-like variant stress testing, uncertainty-aware scoring, and clade-balanced normalized coverage ranking.

The rolling retrospective evaluation showed that FluEvoFormer improved held-out HI antigenicity prediction for the available historical A/H1N1 and A/H3N2 corpora, while the post-2012 H1N1 dominance and vaccine-ranking evaluation specifically concerned A(H1N1)pdm09. The model achieved a lower MAE and RMSE than the HA-only MSA-transformer baseline and produced a better calibration. The temporal dominance module also improved the future strain-distribution prediction, with a lower KL divergence and a higher Recall@20 than the last-season dominance, the static protein language model, and other static scoring baselines.

The vaccine-ranking results showed that FluEvoFormer selected candidates with higher normalized empirical coverage scores in seven out of 10 A(H1N1)pdm09 seasons and nine out of 10 A/H3N2 seasons. The gains were stronger for A/H3N2, where antigenic drift and clade replacement create a greater need for prospective modeling. The 2019–2020 A/H3N2 case study further showed that the proposed ranking provided a broader predicted coverage across the 3C.2a1b.1a/b and 3C.2a1b.2b/a clusters than the historical recommendation.

The ablation study confirmed that the full model benefited from the combined contribution of HA–NA fusion, structure-guided residue learning, temporal dominance modeling, generated future-like stress testing, uncertainty adjustments, and clade-balanced scoring. The clinical correlation analysis showed a positive association between the predicted normalized coverage score and external vaccine-effectiveness and disease-burden indicators, although these correlations should be interpreted as supportive evidence rather than direct clinical proof.

Overall, FluEvoFormer provides a computational decision-support framework for influenza vaccine candidate prioritization. The model can narrow the candidate space, estimate future antigenic coverage, quantify the prediction uncertainty, highlight the clade-level mismatch risk, and provide interpretable residue-level signals for downstream experimental evaluation. Future work should validate the framework prospectively across new influenza seasons, expand multi-laboratory antigenicity data, improve the region-aware surveillance correction, and assess integration with routine public health vaccine-strain-selection workflows. A separate extension should evaluate B/Victoria and B/Yamagata using lineage-specific sequence, antigenicity, temporal prevalence, vaccine-composition, and structural datasets, followed by independent rolling retrospective validation.

## Figures and Tables

**Table 1:** Data sources used in the proposed study.

Dataset Group	Source	Main Variables	Use in This Study
Sequence data	GISAID/NCBI Influenza Virus Database [23,24]	HA sequence, NA sequence, strain name, collection date, subtype, region, host, passage history	Dominance prediction, sequence pretraining, clade analysis, HA–NA fusion
Antigenicity data	WHO Collaborating Centre HI reports, including Francis Crick WIC reports [18,25,26]	Vaccine strain, virus strain, HI value, assay date	Vaccine–virus antigenicity prediction and empirical coverage estimation
Vaccine composition	WHO/GISAID human influenza vaccine-composition records [18,27]	Recommended vaccine strain, season, subtype	Historical comparator and candidate validation
Clinical effectiveness	CDC, I-MOVE, SPSN, and published vaccine-effectiveness estimates [18,28,29]	Vaccine effectiveness, subtype, season, disease burden averted	External clinical correlation analysis
Protein structure	Protein Data Bank/AlphaFold predicted structure resources [30,31]	3D coordinates, residue contacts, structural annotations	Structure-guided residue graph learning

**Table 2:** Final analytical dataset used for model training and rolling evaluation.

Data Component	Subtype	Total Processed Records	Training/ Validation Split	Test/Evaluation Design	Seasons Covered	Missing or Excluded Rate	Main Use
HA sequences	A/H1N1	23,736 non-repeated HA sequences	Cutoff-specific 9:1 split	Rolling winter seasons	2012–2021	Seasons with <100 HA samples excluded	Dominance, pretraining
NA sequences	A/H1N1	18,920 matched NA sequences	Cutoff-specific 9:1 split	Matched rolling subset	2012–2021	Missing HA–NA pair excluded	HA–NA fusion
HA sequences	A/H3N2	28,546 non-repeated HA sequences	Cutoff-specific 9:1 split	Rolling winter seasons	2012–2021	Seasons with <100 HA samples excluded	Dominance, pretraining
NA sequences	A/H3N2	21,860 matched NA sequences	Cutoff-specific 9:1 split	Matched rolling subset	2012–2021	Missing HA–NA pair excluded	HA–NA fusion
HI pairs	A/H1N1	70,631 HA-pair records	8:1:1 before each cutoff	Held-out HI-pair test set	2012–2021	58,420 HA–NA matched pairs	Antigenicity
HI pairs	A/H3N2	63,299 HA-pair records	8:1:1 before each cutoff	Held-out HI-pair test set	2012–2021	51,870 HA–NA matched pairs	Antigenicity
Structure graphs	Both	93,062 sequence-mapped graphs	Template-guided mapping	Residue-level analysis	2012–2021	Template coverage not uniform across all residues	Graph learning

Note: The historical A/H1N1 source corpus includes both pre-2009 seasonal A/H1N1 and A(H1N1)pdm09 records. The results derived from the combined historical sequence or HI corpus are labeled A/H1N1, whereas the target-season dominance and vaccine-ranking results for 2012–2013 through 2021–2022 refer to A(H1N1)pdm09.

**Table 3:** Antigenicity prediction performance on held-out vaccine–virus HI pairs.

Model	Subtype	MAE	RMSE	Pearson *r*	Spearman ρ	Calibration Error	Rank
BLOSUM-nearest HI baseline	A/H1N1	0.566	0.761	0.61	0.58	0.151	6
Linear regression + substitutions	A/H1N1	0.532	0.719	0.65	0.62	0.137	5
CNN	A/H1N1	0.496	0.671	0.69	0.66	0.121	4
Transformer	A/H1N1	0.444	0.608	0.74	0.71	0.101	3
HA-only MSA-transformer baseline	A/H1N1	0.417	0.574	0.78	0.75	0.084	2
FluEvoFormer	A/H1N1	0.389	0.548	0.80	0.77	0.067	1
BLOSUM-nearest HI baseline	A/H3N2	0.648	0.861	0.57	0.54	0.168	6
Linear regression + substitutions	A/H3N2	0.612	0.824	0.60	0.57	0.153	5
CNN	A/H3N2	0.579	0.776	0.64	0.61	0.136	4
Transformer	A/H3N2	0.517	0.704	0.69	0.66	0.116	3
HA-only MSA-transformer baseline	A/H3N2	0.489	0.668	0.72	0.69	0.098	2
FluEvoFormer	A/H3N2	0.456	0.631	0.75	0.72	0.081	1

**Table 4:** Future dominance prediction performance across rolling seasons.

Model	Subtype	KL Divergence	RMSE	Recall@10	Recall@20	Spearman ρ	Rank
Last-season dominance	A/H1N1	0.492	0.058	0.44	0.58	0.65	5
CSCS-style static score	A/H1N1	0.428	0.052	0.50	0.64	0.60	4
EVEscape-style static score	A/H1N1	0.401	0.049	0.53	0.67	0.52	3
Static protein LM	A/H1N1	0.354	0.046	0.57	0.70	0.70	2
FluEvoFormer	A/H1N1	0.255	0.036	0.69	0.81	0.79	1
Last-season dominance	A/H3N2	0.611	0.071	0.37	0.51	0.69	5
CSCS-style static score	A/H3N2	0.542	0.065	0.43	0.57	0.67	4
EVEscape-style static score	A/H3N2	0.501	0.061	0.47	0.61	0.61	3
Static protein LM	A/H3N2	0.462	0.057	0.50	0.64	0.71	2
FluEvoFormer	A/H3N2	0.289	0.043	0.65	0.80	0.83	1

**Table 5:** Season-wise vaccine candidate ranking performance using normalized empirical coverage score.

Season	Subtype	Historical Recommended Strain	FluEvoFormer Top-Ranked Strain	Historical Empirical NCS	Proposed Empirical NCS	Difference	Winner
2012–2013	A/H1N1	Rec-H1N1-2012	FEF-H1N1-2012-A	0.744	0.771	+0.027	Proposed
2012–2013	A/H3N2	Rec-H3N2-2012	FEF-H3N2-2012-A	0.638	0.681	+0.043	Proposed
2013–2014	A/H1N1	Rec-H1N1-2013	FEF-H1N1-2013-A	0.701	0.729	+0.028	Proposed
2013–2014	A/H3N2	Rec-H3N2-2013	FEF-H3N2-2013-A	0.604	0.648	+0.044	Proposed
2014–2015	A/H1N1	Rec-H1N1-2014	FEF-H1N1-2014-A	0.786	0.807	+0.021	Proposed
2014–2015	A/H3N2	Rec-H3N2-2014	FEF-H3N2-2014-A	0.582	0.638	+0.056	Proposed
2015–2016	A/H1N1	Rec-H1N1-2015	FEF-H1N1-2015-A	0.732	0.724	−0.008	Historical
2015–2016	A/H3N2	Rec-H3N2-2015	FEF-H3N2-2015-A	0.647	0.693	+0.046	Proposed
2016–2017	A/H1N1	Rec-H1N1-2016	FEF-H1N1-2016-A	0.769	0.801	+0.032	Proposed
2016–2017	A/H3N2	Rec-H3N2-2016	FEF-H3N2-2016-A	0.612	0.668	+0.056	Proposed
2017–2018	A/H1N1	Rec-H1N1-2017	FEF-H1N1-2017-A	0.821	0.816	−0.005	Historical
2017–2018	A/H3N2	Rec-H3N2-2017	FEF-H3N2-2017-A	0.694	0.687	−0.007	Historical
2018–2019	A/H1N1	Rec-H1N1-2018	FEF-H1N1-2018-A	0.754	0.783	+0.029	Proposed
2018–2019	A/H3N2	Rec-H3N2-2018	FEF-H3N2-2018-A	0.628	0.674	+0.046	Proposed
2019–2020	A/H1N1	Rec-H1N1-2019	FEF-H1N1-2019-A	0.793	0.787	−0.006	Historical
2019–2020	A/H3N2	Rec-H3N2-2019	FEF-H3N2-2019-A	0.661	0.701	+0.040	Proposed
2020–2021	A/H1N1	Rec-H1N1-2020	FEF-H1N1-2020-A	0.682	0.739	+0.057	Proposed
2020–2021	A/H3N2	Rec-H3N2-2020	FEF-H3N2-2020-A	0.556	0.620	+0.064	Proposed
2021–2022	A/H1N1	Rec-H1N1-2021	FEF-H1N1-2021-A	0.748	0.774	+0.026	Proposed
2021–2022	A/H3N2	Rec-H3N2-2021	FEF-H3N2-2021-A	0.617	0.659	+0.042	Proposed

**Table 6:** Overall vaccine ranking performance compared with baseline strategies.

Method	Subtype	Mean Empirical NCS	Median Empirical NCS	Win Rate	Best Tested- Strain Rate	Mean Rank of Selected Strain
Last-season dominance	A/H1N1	0.733	0.735	0.50	0.20	3.1
Antigenicity-only score	A/H1N1	0.754	0.759	0.60	0.40	2.6
Dominance + antigenicity score	A/H1N1	0.766	0.771	0.70	0.60	2.1
FluEvoFormer	A/H1N1	0.773	0.778	0.70	0.70	1.6
Last-season dominance	A/H3N2	0.600	0.602	0.40	0.10	3.8
Antigenicity-only score	A/H3N2	0.633	0.636	0.55	0.30	3.0
Dominance + antigenicity score	A/H3N2	0.654	0.655	0.70	0.50	2.3
FluEvoFormer	A/H3N2	0.667	0.671	0.90	0.60	1.7

**Table 7:** Correlation between predicted normalized coverage score, vaccine effectiveness, and disease burden indicators.

Outcome	Data Source	Subtype	Pearson *r*	Spearman ρ	*p*-Value
Vaccine effectiveness	CDC	Combined A/H1N1+A/H3N2	0.872	0.903	0.0011
Vaccine effectiveness	I-MOVE	Combined A/H1N1+A/H3N2	0.801	0.842	0.0090
Vaccine effectiveness	SPSN	Combined A/H1N1+A/H3N2	0.742	0.812	0.0180
Averted illnesses	CDC	Combined weighted score	0.692	0.683	0.0410
Averted medical visits	CDC	Combined weighted score	0.704	0.697	0.0340

**Table 8:** Ablation study of FluEvoFormer components.

Model Variant	HI MAE	Dominance KL	Recall@20	Mean Empirical NCS	Win Rate	Performance Drop
Full FluEvoFormer	0.423	0.272	0.805	0.720	0.80	Reference
Without NA encoder	0.438	0.288	0.786	0.710	0.75	−1.4% NCS
Without structure graph	0.447	0.291	0.779	0.708	0.75	−1.7% NCS
Without diffusion simulator	0.429	0.305	0.753	0.703	0.70	−2.4% NCS
Without uncertainty penalty	0.425	0.276	0.801	0.709	0.70	−1.5% NCS
Without clade-balanced score	0.426	0.280	0.793	0.711	0.75	−1.3% NCS
HA-only MSA-transformer backbone	0.453	0.319	0.741	0.695	0.70	−3.5% NCS
Sequence-only transformer	0.462	0.336	0.722	0.687	0.65	−4.6% NCS

**Table 9:** Uncertainty calibration and reliability analysis.

Model	Subtype	ECE	95% Coverage	NLL	Risk-Adjusted Win Rate
Transformer baseline	A/H1N1	0.096	0.881	1.214	0.60
FluEvoFormer without uncertainty	A/H1N1	0.074	0.907	1.061	0.65
FluEvoFormer	A/H1N1	0.061	0.939	0.902	0.70
Transformer baseline	A/H3N2	0.111	0.856	1.382	0.55
FluEvoFormer without uncertainty	A/H3N2	0.083	0.892	1.187	0.75
FluEvoFormer	A/H3N2	0.071	0.934	1.012	0.90

**Table 10:** Evaluation of generated future-like variant pools used for model-based vaccine candidate stress testing.

Season	Subtype	Generated Pool Size	Novelty Rate	Clade Consistency	Future-Neighbor Recall	Plausibility Filter Pass Rate	Effect on Predicted NCS
2018–2019	A/H1N1	1500	0.31	0.88	0.63	0.95	+0.010
2018–2019	A/H3N2	2000	0.42	0.82	0.58	0.91	+0.019
2019–2020	A/H1N1	1500	0.29	0.89	0.65	0.95	+0.009
2019–2020	A/H3N2	2000	0.44	0.80	0.57	0.90	+0.022
2020–2021	A/H1N1	1200	0.27	0.86	0.60	0.94	+0.012
2020–2021	A/H3N2	1500	0.40	0.79	0.55	0.89	+0.025
2021–2022	A/H1N1	1400	0.30	0.88	0.62	0.94	+0.011
2021–2022	A/H3N2	1800	0.41	0.81	0.56	0.90	+0.021

**Table 11:** Residue-level explainability results for antigenicity and dominance prediction.

Subtype	Protein	Residue/ Site	Mutation Pattern	Biological/ Structural Context	Attribution Score	Structural Neighborhood	Interpretation
A/H1N1	HA	HA-156/158	Substitution cluster	HA antigenic head region	0.82	Receptor-binding proximal residues	Main contribution to antigenic-distance prediction
A/H1N1	HA	HA-190/193	Substitution cluster	Receptor-binding and antigenic interface	0.77	Head-domain contact pocket	Linked with vaccine–virus match variation
A/H1N1	NA	NA-151/153	Local substitution signal	NA surface loop	0.58	NA head-domain surface contacts	Putative secondary model signal associated with dominance; experimental role not established
A/H3N2	HA	HA-145/159	Substitution and glycosylation-associated signal	HA antigenic head region	0.88	High-contact head-domain neighborhood	Strong model attribution near the HA antigenic head; experimental causality not established
A/H3N2	HA	HA-186/189	Substitution cluster	Receptor-binding proximal region	0.83	Antigenic-site contact shell	Strong influence on pairwise HI prediction
A/H3N2	NA	NA-245/248	Surface substitution signal	NA functional surface region	0.61	NA head-domain neighborhood	Secondary signal for dominance and dual-protein fitness

**Table 12:** Paired season-wise statistical comparison between FluEvoFormer-ranked candidates and historical vaccine recommendations.

Comparison	Metric	Subtype	Mean Paired Difference	Positive Seasons	Test	Raw *p*-Value	BH-Adjusted *q*-Value	Significant
FluEvoFormer vs. historical recommendation	Empirical NCS	A(H1N1)pdm09	+0.020	7/10	Exact one-sided Wilcoxon signed-rank test	0.0137	0.0137	Yes
FluEvoFormer vs. historical recommendation	Empirical NCS	A/H3N2	+0.043	9/10	Exact one-sided Wilcoxon signed-rank test	0.0020	0.0039	Yes

**Table 13:** Computational efficiency of the proposed framework.

Model	Training Time	Inference Time/Season	GPU Memory	Pairs Screened/Min
CNN baseline	5.8 h	1.6 min	9.2 GB	18,400
Transformer baseline	16.4 h	4.1 min	19.8 GB	10,600
HA-only MSA-transformer baseline	24.7 h	6.9 min	26.4 GB	7400
FluEvoFormer	54.8 h	8.7 min	38.6 GB	5800

## Data Availability

Influenza sequence records and associated metadata were obtained from GISAID and the NCBI Influenza Virus Database. GISAID-derived accession identifiers and associated metadata will be shared only in accordance with the GISAID access terms. HI antigenicity data were obtained from WHO Collaborating Centre reports, including the Worldwide Influenza Centre annual and interim reports from the Francis Crick Institute. Human influenza vaccine-composition records were obtained from WHO/GISAID vaccine-composition resources. Vaccine-effectiveness estimates were obtained from the US Centers for Disease Control and Prevention (CDC), the Influenza Monitoring Vaccine Effectiveness in Europe (I-MOVE) network, and the Canadian Sentinel Practitioner Surveillance Network (SPSN), as compiled and documented by Shi et al. [18]. CDC disease-burden indicators were obtained from the corresponding CDC resources [28,29]. Protein structure templates and predicted structures were obtained from the Protein Data Bank and AlphaFold structure resources. The research-related files generated for this study, including the trained model checkpoint, hyperparameter configuration, training-metric log, and evaluation-summary files, are hosted on Zenodo at https://doi.org/10.5281/zenodo.20711915. The deposited files include fluevoformer_best_weights.pt, hyperparameters.json, training_metrics.csv, and evaluation_summary.json. Restricted database records, restricted metadata, and generated viral sequence content are not included in the public deposit.

## Supplementary Materials

The following supporting information can be downloaded at: https://www.mdpi.com/article/10.3390/v18080843/s1, Supplementary Methods: Complete mathematical formulation, component-level equations, loss functions, training procedures, ranking procedures, and implementation details; Figure S1: Temporal dominance prediction module; Figure S2: Controlled future-like variant simulator; Figure S3: Vaccine–virus antigenicity and uncertainty predictor; Figure S4: Computational efficiency and deployment trade-off; Table S1: Sequence preprocessing counts; Table S2: HI-pair preprocessing and matching counts; Table S3: Residue-contact graph-construction characteristics; Table S4: Season-wise rolling retrospective evaluation design, candidate windows, and evaluation labels; Table S5: Baseline model inputs and comparison purposes; Table S6: Architecture settings, optimization parameters, training duration, and implementation values; Table S7: Coded strain identifiers and reporting basis for historical and FluEvoFormer-ranked candidates; Algorithm S1: Cutoff-restricted FluEvoFormer training procedure; Algorithm S2: Uncertainty-adjusted and clade-balanced vaccine candidate ranking procedure.
