# Supplementary material for: FluEvoFormer: A Structure-Guided Generative Foundation Model for Prospective Influenza Antigenic Evolution and Vaccine Strain Selection
Source: Viruses. 2026 Aug 1;18(8):843. doi: 10.3390/v18080843 (PMC13517461; doi:10.3390/v18080843)
Supplement: Supplementary file 1 [file viruses-18-00843-s001.zip › viruses-4419909-supplementary.pdf]

Supplementary Materials

The supplementary material provides the complete mathematical formulation of FluEvoFormer, detailed training and vaccine-ranking algorithms, expanded data-processing summaries, the complete rolling retrospective evaluation design, baseline-model descriptions, implementation and hyperparameter settings, module-specific architecture figures, candidate-code definitions, and computational-efficiency results. These materials support methodological reproducibility while allowing the main text to emphasize the biological rationale, evaluation design, and principal findings.

Supplementary Methods: Mathematical Formulation and Implementation Details

Problem Formulation and Antigenicity Labels

The study formulates influenza vaccine strain selection as a prospective ranking problem. Let x_i denote a viral isolate, v denote a candidate vaccine strain, and T denote the target influenza season. Each viral isolate is represented as:

$$x_i = \{s_i^{HA}, s_i^{NA}, t_i, r_i, c_i, q_i, G_i^{HA}, G_i^{NA}\}, \tag{S1}$$

where s_i^{HA} and s_i^{NA} denote the hemagglutinin and neuraminidase amino acid sequences, t_i denotes the collection time, r_i denotes the geographical region, c_i denotes the subtype or clade label, q_i denotes additional metadata, and G_i^{HA} and G_i^{NA} denote residue-contact graphs. The task is to estimate the future dominance of each viral isolate and the antigenic match between each candidate vaccine strain and the future circulating viral population.

A vaccine–virus antigenicity pair is represented as:

$$(v, x, y_{v,x}), \tag{S2}$$

where $y_{v,x}$ is the antigenicity label derived from hemagglutination inhibition assay measurements. Following established antigenic phenotype modeling, the relative antigenic similarity is calculated as:

$$y_{v,x} = \min(\log_2 HI(v,x) - \log_2 HI(v,v), 0), \tag{S3}$$

where $HI(v,x)$ denotes the hemagglutination inhibition value between vaccine strain v and virus strain x, and $HI(v,v)$ denotes the homologous hemagglutination inhibition value of the vaccine reference strain. Values closer to zero indicate stronger antigenic similarity. Because Eq. (S3) gives non-positive values, the model converts the predicted antigenicity into a bounded antigenic match score:

$$A(v,x) = \exp\left(\frac{y_{v,x}}{\tau_{HI}}\right), \tag{S4}$$

where τ_{HI} is a validation-selected scaling parameter. This transformation gives $A(v,x) \in (0,1]$, where larger values indicate better predicted antigenic match.

Sequence Alignment, Clade Annotation, and Structural Mapping

For each target season, only records available before the vaccine-selection cutoff were included in model development. Sequence matching and protein alignment were performed with protein sequence search and alignment tools, and standardized viral clade assignment was used for subtype-specific clade annotation. Experimental structures were used when available and were supplemented with predicted structure resources for sequence-mapped residue-contact graph construction.

Sequence Preprocessing and Seasonal Dominance Estimation

Raw hemagglutinin and neuraminidase sequences are filtered to remove incomplete records, non-human host records, uncertain subtype labels, abnormal sequence lengths, missing collection dates, and duplicated metadata errors. Duplicate protein sequences within each season are collapsed, but their occurrence counts are retained because these counts define seasonal dominance. Matched hemagglutinin–neuraminidase records are retained when both proteins are available for the same isolate or strain record. Sequence alignment is performed by subtype and protein to preserve position-specific mutation information.

Each winter evaluation season is defined from October to March. For each target season T, sequence records collected before the corresponding cutoff date are used for training, while the target season is reserved for retrospective evaluation. The observed seasonal dominance of a viral isolate x_i is calculated as:

$$p_T(x_i) = \frac{n_T(x_i)}{\sum_{j=1}^{N_T} n_T(x_j)}, \tag{S5}$$

where $n_T(x_i)$ is the frequency of sequence x_i in season T, and N_T is the number of unique sequences observed in that season. This value represents the empirical probability that a sequence occurs in the circulating viral population during season T.

Antigenicity Pair Construction

The antigenicity dataset contains vaccine–virus pairs with available hemagglutination inhibition measurements. Strain names from hemagglutination inhibition reports are matched to sequence records using strain identifiers, collection metadata, and subtype labels. Pairs without reliable sequence matches are excluded. If several sequence records map to the same strain name, all valid sequence-level mappings are retained during pair construction. If multiple hemagglutination inhibition values exist for the same vaccine–virus pair, their geometric mean is used before log-scale transformation. This reduces the effect of assay-level repetition while preserving the fold-dilution structure of hemagglutination inhibition data.

For each cutoff-specific training period, only vaccine–virus pairs with both sequences available before the cutoff date are used to train the antigenicity predictor. The pair-level dataset is split into training, validation, and held-out test subsets under the same temporal restriction. This procedure ensures that future hemagglutination inhibition information does not influence predictions for the target season.

Protein Structure Graph Construction

For each available hemagglutinin and neuraminidase protein structure, a residue-contact graph is constructed:

$$G = (V, E), \tag{S6}$$

where each node $v_k \in V$ represents an amino acid residue and each edge $e_{kl} \in E$ represents structural contact between residues k and l. Edges are assigned using a distance threshold:

$$e_{kl} = \begin{cases} 1, & d(k,l) < \delta, \\ 0, & \text{otherwise}, \end{cases} \tag{S7}$$

where $d(k,l)$ is the Euclidean distance between residues k and l, and δ is the contact threshold. The contact threshold was set to 8 Å throughout the analysis.

Each residue node stores amino acid identity, aligned position, conservation score, antigenic-site indicator, glycosylation-site indicator, solvent-accessibility proxy, and pre-trained protein embedding. Structure templates are mapped to sequence positions after subtype-specific alignment. This representation allows the model to capture structural relationships between residues that may be distant in the linear sequence but close in the folded protein.

Dual HA–NA Protein Sequence Encoder

The model represents each isolate through a joint hemagglutinin–neuraminidase input sequence:

$$S_i = [CLS]\ s_i^{HA}\ [SEP]\ s_i^{NA}\ [META]. \tag{S8}$$

The input embedding combines amino acid identity, residue position, collection time, and metadata:

$$E_i = Emb(S_i) + Pos(S_i) + Time(t_i) + Meta(q_i). \tag{S9}$$

A transformer encoder then produces contextual residue embeddings:

$$H_i = Transformer(E_i). \tag{S10}$$

The transformer architecture is used because self-attention can learn long-range residue dependencies and pairwise context across viral protein sequences. Separate segment embeddings distinguish hemagglutinin, neuraminidase, and metadata tokens, which allows the model to learn protein-specific and cross-protein mutation patterns.

Structure-Aware Graph Encoder

The structure-aware module updates residue embeddings using residue-contact graphs. The graph encoder follows an attention-based neighborhood aggregation strategy:

$$h_k^{(l+1)} = \sigma\left(W_1 h_k^{(l)} + \sum_{j \in \mathcal{N}(k)} \alpha_{kj} W_2 h_j^{(l)}\right), \tag{S11}$$

where $h_k^{(l)}$ is the representation of residue k at layer l, $\mathcal{N}(k)$ is the structural neighborhood of residue k, α_{kj} is the learned attention weight between residues k and j, and $\sigma(\cdot)$ is a nonlinear activation function. This design allows each residue to receive information from structurally nearby residues instead of relying only on adjacent sequence positions. The resulting structure-aware representation is denoted as ${H^{struct}}_i$

HA–NA Cross-Fusion Module

The hemagglutinin–neuraminidase cross-fusion module transfers information between the two surface proteins. The hemagglutinin representation attends to the neuraminidase representation as:

$$Z_i^{HA \leftarrow NA} = Attention(Q = H_i^{HA}, K = H_i^{NA}, V = H_i^{NA}), \tag{S12}$$

and the neuraminidase representation attends to the hemagglutinin representation as:

$$Z_i^{NA \leftarrow HA} = Attention(Q = H_i^{NA}, K = H_i^{HA}, V = H_i^{HA}). \tag{S13}$$

The final viral embedding is obtained by pooling sequence-level, cross-protein, and structure-aware features:

$$z_i = Pool([Z_i^{HA \leftarrow NA}; Z_i^{NA \leftarrow HA}; H_i^{struct}]). \quad \text{(S14)}$$

This embedding summarizes the viral isolate and is used by the dominance predictor, antigenicity predictor, diffusion module, and candidate ranking score.

Temporal Dominance Predictor

The temporal module predicts how the dominance of a viral sequence changes across seasons. Instead of treating viral fitness as static, the module models the unnormalized frequency $n_i(t)$ of strain x_i through a neural growth-rate function:

$$\frac{dn_i(t)}{dt} = r_\theta(z_i, t) n_i(t), \quad \text{(S15)}$$

where $r_\theta(z_i, t)$ is a learned time-dependent growth rate. This formulation follows the idea that strain dominance changes as viral populations compete over time. The model maps the viral embedding and target season into a scalar dominance logit $g_\theta(z_i, T)$. Future dominance is then estimated by:

$$\hat{p}_T(x_i) = \frac{\exp(g_\theta(z_i, T))}{\sum_j \exp(g_\theta(z_j, T))}. \quad \text{(S16)}$$

The softmax normalization ensures that predicted dominance values sum to one within the candidate circulating virus pool. Figure S1 illustrates how the temporal module converts cutoff-restricted historical sequence frequencies into future dominance estimates.

Controlled Generative Future-Like Variant Simulator

The generative module performs aggregate computational stress testing by simulating future-like protein variants in embedding space and aligned sequence-token space. The module uses a discrete diffusion process for amino acid tokens, following diffusion model-ing principles for discrete state spaces. A noisy sequence at diffusion step k is sampled as:

$$q(s_k|s_0) = Cat((1 - \beta_k) \cdot onehot(s_0) + \beta_k \cdot U), \quad \text{(S17)}$$

where s_0 is the original sequence, s_k is the corrupted sequence, β_k is the noise schedule, and U is the uniform amino acid distribution. The model learns the reverse denoising distribution:

$$p_\theta(s_{k-1}|s_k, t, c, r), \quad \text{(S18)}$$

where t, c, and r condition the generation process on season, clade, and region. The diffusion loss is:

$$\mathcal{L}_{diff} = -\sum_k \log p_\theta(s_{k-1}|s_k, t, c, r). \quad \text{(S19)}$$

The generated future-like pool is used only to estimate aggregate expected antigenic coverage and to test the robustness of candidate rankings under future-like scenarios. This model-derived coverage estimate is not equivalent to clinical vaccine effectiveness. The manuscript reports only summary-level metrics, embedding distributions, and coverage effects. It does not report generated viral sequences or treat them as laboratory-ready candidates. This restriction keeps the generative component limited to computational prioritization and avoids actionable viral design details, while still allowing the model to estimate coverage under plausible antigenic drift scenarios. Recent protein diffusion studies support the broader feasibility of diffusion-based protein modeling, but all vaccine-relevant outputs require experimental validation before any practical use. Figure S2

summarizes the controlled future-like variant simulator used for aggregate computational stress testing.

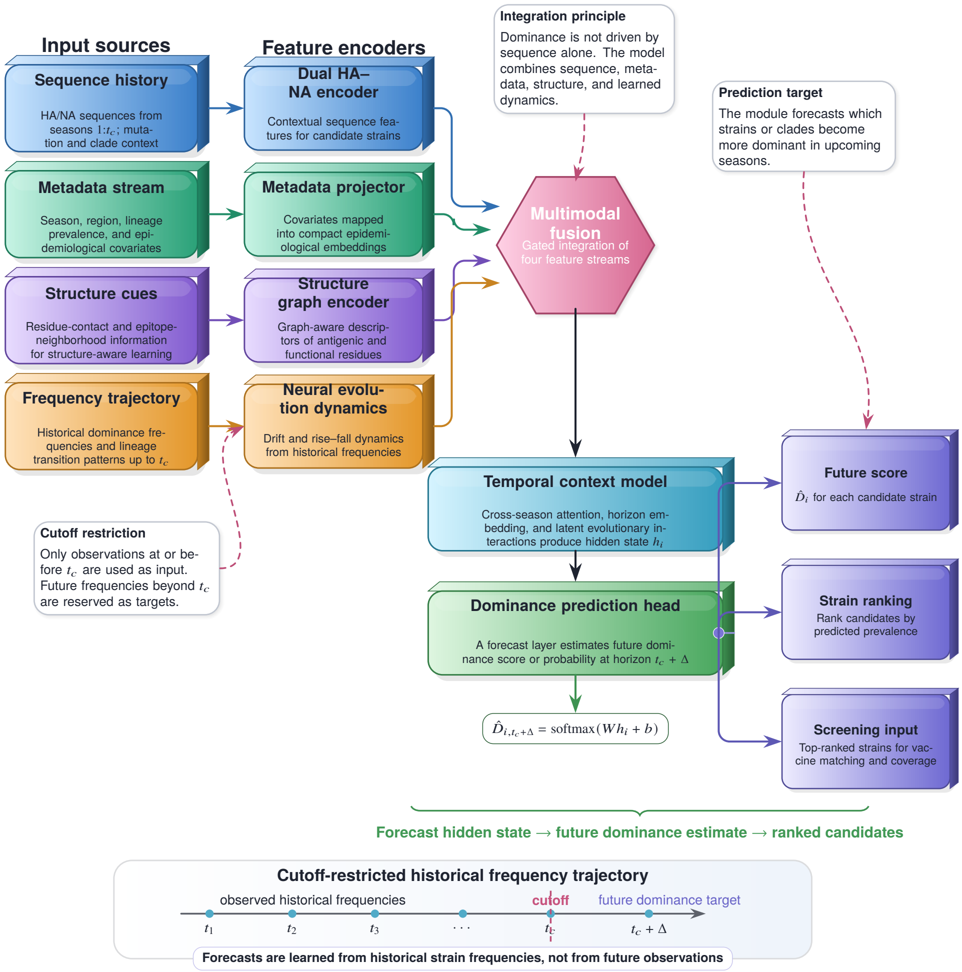

Figure S1. Stepwise temporal dominance prediction module. Sequence history, epidemiological metadata, residue-contact structure, and historical frequency trajectories are encoded separately and combined through multimodal fusion. The temporal context model produces a future dominance probability and ranked strain list. The lower timeline illustrates the cutoff restriction: observations at or before t_c are used as inputs, whereas frequencies after t_c are reserved as prediction targets.

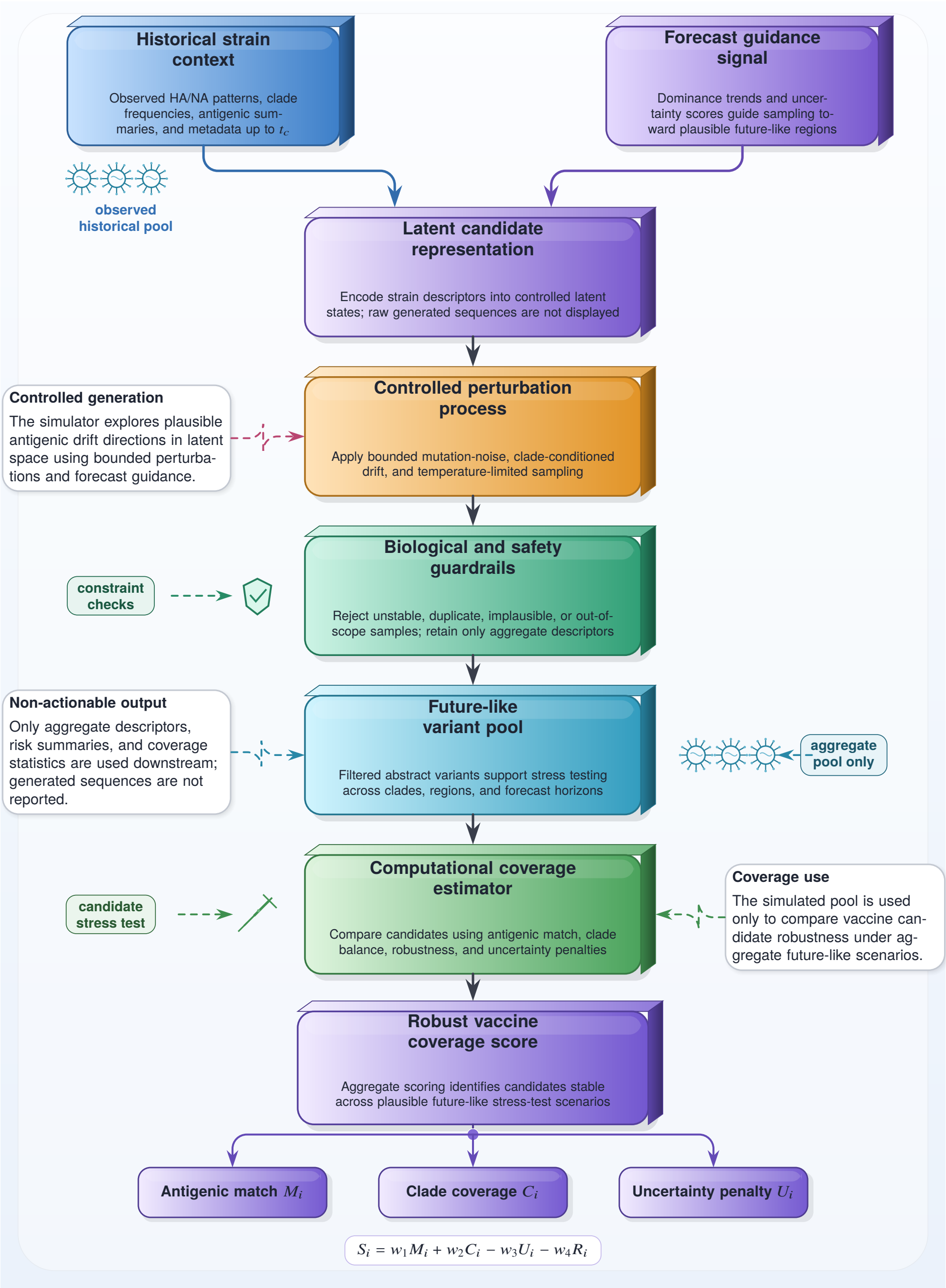

Figure S2. Controlled future-like variant simulation used for model-based vaccine candidate stress testing. Historical HA–NA patterns and forecast guidance are encoded into latent candidate representations. Bounded perturbation, clade conditioning, and biological plausibility filters produce an aggregate future-like pool. Candidate vaccines are then compared using predicted antigenic match, clade coverage, and uncertainty penalties to calculate a robust coverage score. Only aggregate descriptors and coverage statistics are used downstream; generated viral sequences are not reported.

Vaccine–Virus Antigenicity Predictor

For each candidate vaccine strain v and virus strain x, the model constructs a pairwise representation through vaccine–virus cross-attention:

$$h_{v,x} = CrossAttention(z_v, z_x). \tag{S20}$$

The antigenicity head predicts the relative hemagglutination inhibition-derived antigenic similarity:

$$\hat{y}_{v,x} = f_\theta(h_{v,x}). \tag{S21}$$

Because hemagglutination inhibition measurements are noisy and can vary across experiments, the model also predicts input-dependent uncertainty:

$$(\mu_{v,x}, \sigma^2_{v,x}) = f_\theta(h_{v,x}). \tag{S22}$$

The heteroscedastic antigenicity loss is:

$$\mathcal{L}_{HI} = \frac{(y_{v,x} - \mu_{v,x})^2}{2\sigma^2_{v,x}} + \frac{1}{2}\log(\sigma^2_{v,x}). \tag{S23}$$

This loss downweights highly uncertain observations and allows the model to express larger uncertainty for difficult vaccine–virus pairs. The predicted mean $\mu_{v,x}$ is converted into an antigenic match score using Eq. (S4). Figure S3 shows the vaccine–virus antigenicity prediction branch and uncertainty head.

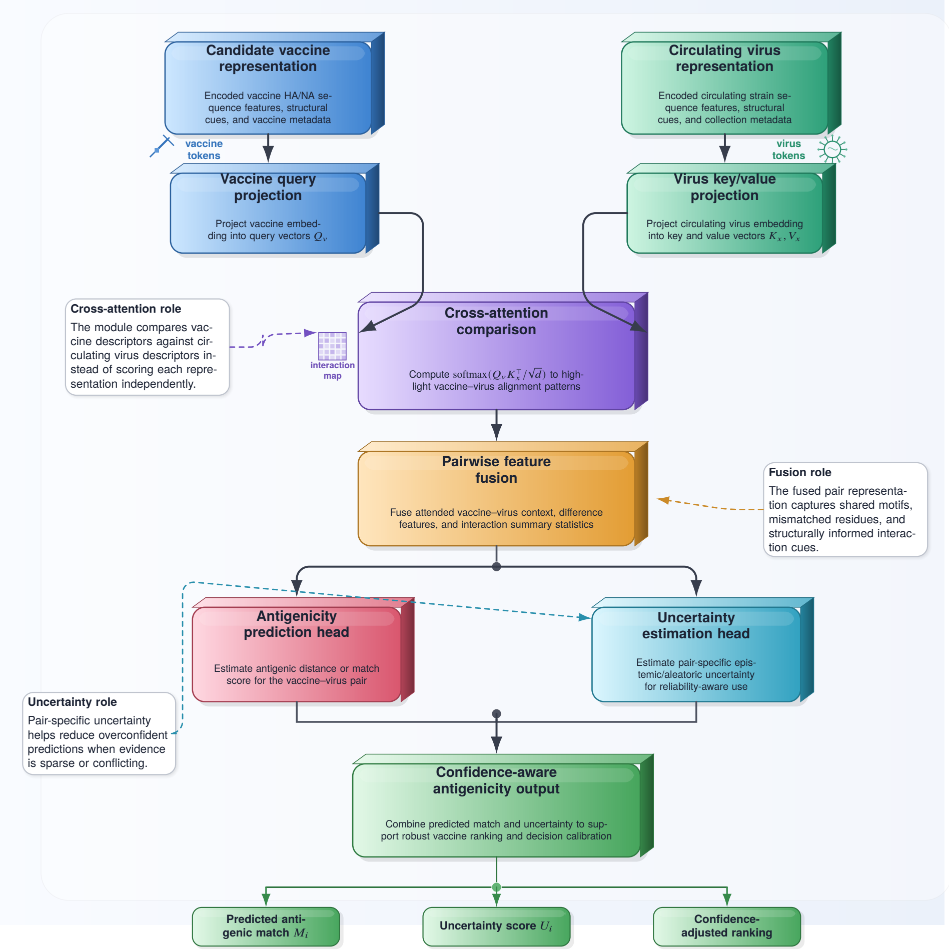

Figure S3. Vaccine–virus antigenicity and uncertainty prediction module. Representations of the candidate vaccine and circulating virus are projected into query, key, and value vectors and compared through cross-attention. Pairwise feature fusion combines shared motifs, residue differences, and structure-informed interaction signals. Separate output heads estimate antigenic match and pair-specific uncertainty, which are combined to support confidence-adjusted vaccine ranking.

Robust Vaccine Coverage Score

For a target season T, the candidate circulating virus set is defined as:

$$\mathcal{X}_T = \mathcal{X}_T^{observed} \cup \mathcal{X}_T^{generated}, \tag{S24}$$

where $\mathcal{X}_T^{observed}$ contains eligible observed viruses and $\mathcal{X}_T^{generated}$ contains only aggregate future-like computational variants. The predicted normalized coverage score of vaccine v is:

$$NCS_T(v) = \sum_{x \in \mathcal{X}_T} \hat{p}_T(x)\hat{A}(v,x), \tag{S25}$$

where $\hat{p}_T(x)$ is predicted dominance and $\hat{A}(v,x)$ is predicted antigenic match. This score measures the expected antigenic coverage of a vaccine against the future circulating virus pool.

The uncertainty term is:

$$U_T(v) = \sum_{x \in \mathcal{X}_T} \hat{p}_T(x)\sigma_{v,x}^2. \tag{S26}$$

The uncertainty-adjusted score is:

$$Score_T(v) = NCS_T(v) - \lambda U_T(v), \tag{S27}$$

where λ controls the penalty assigned to uncertain vaccine–virus predictions. Model calibration is assessed on validation seasons using reliability diagrams and expected calibration error, following standard calibration practice for neural networks.

To avoid selecting a candidate that performs well only against one dominant clade while poorly covering other relevant clades, the clade-balanced coverage term is defined as:

$$NCS_T^{min}(v) = \min_{k \in K} \sum_{x \in C_k} \hat{p}_T(x)\hat{A}(v,x), \tag{S28}$$

where K denotes the clade set and C_k denotes viruses belonging to clade k. The final robust vaccine score is:

$$RScore_T(v) = (1-\alpha)Score_T(v) + \alpha NCS_T^{min}(v), \tag{S29}$$

where α controls the influence of clade-balanced coverage. Candidate vaccines are ranked in descending order of $RScore_T(v)$.

For retrospective evaluation, the empirical normalized coverage score is computed using observed seasonal dominance and experimental hemagglutination inhibition values:

$$eNCS_T(v) = \sum_{x \in \mathcal{X}_T^{observed}} p_T(x)A_{HI}(v,x), \tag{S30}$$

where $p_T(x)$ is observed dominance and $A_{HI}(v,x)$ is the antigenic match score calculated from measured hemagglutination inhibition data. The empirical score is used only after the target season has occurred and serves as the main retrospective surrogate for evaluating candidate vaccine ranking.

Multi-Task Training Objective

The model is optimized using a multi-task objective:

$$\mathcal{L}_{total} = \lambda_1\mathcal{L}_{MLM} + \lambda_2\mathcal{L}_{diff} + \lambda_3\mathcal{L}_{dom} + \lambda_4\mathcal{L}_{HI} + \lambda_5\mathcal{L}_{clade} + \lambda_6\mathcal{L}_{struct} + \lambda_7\mathcal{L}_{cal}. \tag{S31}$$

Here, $\mathcal{L}_{MLM}$ trains masked amino acid recovery, $\mathcal{L}_{diff}$ trains the future-like variant simulator, $\mathcal{L}_{dom}$ trains future dominance prediction, $\mathcal{L}_{HI}$ trains antigenicity prediction, $\mathcal{L}_{clade}$ trains subtype or clade recognition, $\mathcal{L}_{struct}$ trains structure-aware residue consistency, and $\mathcal{L}_{cal}$ improves uncertainty calibration.

The dominance loss is:

$$\mathcal{L}_{dom} = -\sum_{(x_i,T)} \log \hat{p}_T(x_i). \tag{S32}$$

The clade classification loss is:

$$\mathcal{L}_{clade} = -\sum_i c_i \log \hat{c}_i. \tag{S33}$$

The calibration loss is:

$$\mathcal{L}_{cal} = \left|P(y \in \hat{I}_{95}) - 0.95\right|, \tag{S34}$$

where $\hat{I}_{95}$ is the predicted 95% interval. The task weights $\lambda_1, \ldots, \lambda_7$ are selected on validation seasons and kept fixed for test-season evaluation.

Training Strategy

Training is performed in four stages. First, self-supervised pretraining teaches the dual hemagglutinin–neuraminidase encoder general influenza sequence patterns through masked amino acid prediction and sequence-context recovery. Second, supervised antigenicity fine-tuning trains the vaccine–virus cross-attention head using cutoff-restricted hemagglutination inhibition pairs. Third, temporal dominance training learns future seasonal frequency shifts from observed sequence counts. Fourth, joint multi-task fine-tuning aligns antigenicity, dominance, structure, clade, diffusion, and uncertainty objectives. Validation loss is used for checkpoint selection, and test-season data remain unseen during model training. Algorithm S1 summarizes the complete FluEvoFormer training procedure.

Algorithm S1 FluEvoFormer training procedure

Require: HA/NA sequences, metadata, HI pairs, vaccine records, protein structures
Ensure: Trained FluEvoFormer model

1: Clean and align HA/NA sequences by subtype and season.
2: Match HA and NA records where paired surface-protein information is available.
3: Construct seasonal dominance labels from observed sequence frequencies.
4: Build HA and NA residue-contact graphs from structural coordinates.
5: Pretrain the dual HA–NA encoder using masked amino acid prediction.
6: Train the structure-aware graph encoder using residue-contact consistency.
7: Fine-tune the antigenicity predictor using vaccine–virus HI pairs.
8: Train the temporal evolution module using rolling seasonal dominance labels.
9: Train the diffusion module using controlled sequence corruption and denoising.
10: Jointly fine-tune all modules using Eq. (S31).
11: Calibrate uncertainty estimates on validation seasons.

Vaccine Candidate Ranking Algorithm

Supplementary Algorithm S2 converts the model outputs into a ranked list of vaccine candidates. The procedure has four stages. First, the observed and generated future-like virus sets are combined, and a future dominance weight is predicted for every virus. Second, each candidate vaccine is compared with every virus to estimate antigenic match and pair-specific uncertainty. Third, the expected coverage, uncertainty penalty, and minimum clade-specific coverage are calculated. Finally, the candidates are ordered using

the uncertainty-adjusted and clade-balanced robust score. The resulting ranking is intended for downstream experimental prioritization.

Algorithm S2 Stepwise FluEvoFormer vaccine candidate ranking procedure

Require: Trained model; target season T; eligible vaccine candidates $\mathcal{V}_T$; observed virus set $\mathcal{X}_T^{\text{observed}}$; generated future-like virus set $\mathcal{X}_T^{\text{generated}}$; uncertainty weight λ; clade-balance weight α

Ensure: Ranked vaccine candidates with predicted coverage and uncertainty information

Combine the virus sets:

$$\mathcal{X}_T \leftarrow \mathcal{X}_T^{\text{observed}} \cup \mathcal{X}_T^{\text{generated}}$$

for each virus $x \in \mathcal{X}_T$ **do**

  Predict its target-season dominance weight $\hat{p}_T(x)$.

end for

Normalize the dominance weights so that $\sum_{x \in \mathcal{X}_T} \hat{p}_T(x) = 1$.

for each vaccine candidate $v \in \mathcal{V}_T$ **do**

  Set expected coverage $NCS_T(v) \leftarrow 0$.

  Set weighted uncertainty $U_T(v) \leftarrow 0$.

  for each virus $x \in \mathcal{X}_T$ **do**

    Predict antigenic match $\hat{A}(v, x)$.

    Predict pair-specific uncertainty $\sigma^2_{v,x}$.

    Update expected coverage:

$$NCS_T(v) \leftarrow NCS_T(v) + \hat{p}_T(x)\hat{A}(v, x)$$

    Update weighted uncertainty:

$$U_T(v) \leftarrow U_T(v) + \hat{p}_T(x)\sigma^2_{v,x}$$

  end for

  Compute the uncertainty-adjusted score:

$$Score_T(v) \leftarrow NCS_T(v) - \lambda U_T(v)$$

  for each relevant viral clade C_k **do**

    Compute clade-specific expected coverage:

$$Coverage_{T,k}(v) \leftarrow \sum_{x \in C_k} \hat{p}_T(x)\hat{A}(v, x)$$

  end for

  Set the minimum clade coverage:

$$NCS_T^{\min}(v) \leftarrow \min_k Coverage_{T,k}(v)$$

  Compute the final robust score:

$$RScore_T(v) \leftarrow (1 - \alpha)Score_T(v) + \alpha NCS_T^{\min}(v)$$

end for

Sort all vaccine candidates in descending order of $RScore_T(v)$.

Return the ranked candidates together with $NCS_T(v)$, $U_T(v)$, $NCS_T^{\min}(v)$, and $RScore_T(v)$.

Statistical and Calibration Analysis

Prediction performance is evaluated on cutoff-restricted held-out data and rolling retrospective seasons. Antigenicity prediction is assessed with mean absolute error, root mean squared error, Pearson correlation, Spearman rank correlation, and calibration error.

Dominance prediction is assessed with distributional error, top-k recall, and correlation with empirical coverage. Vaccine ranking is assessed using empirical normalized coverage score, season-wise win rate, best-tested-strain rate, and mean rank of the selected strain. Correlation analyses use Pearson correlation when normality assumptions are satisfied and Spearman rank correlation otherwise. Paired season-wise comparisons use Wilcoxon signed-rank testing, and multiple comparisons are adjusted using the Benjamini–Hochberg false discovery rate procedure. Calibration is assessed with expected calibration error, 95% interval coverage, negative log-likelihood, and reliability diagrams.

Baseline Models

The proposed FluEvoFormer model was compared with statistical, machine learning, deep learning, and scoring-based baselines. The last-season dominance baseline used the observed frequency of strains from the preceding season. The clade-frequency and phylogenetic baselines used clade trends and tree-position information to approximate future expansion. Conventional machine learning baselines included random forest and XGBoost models trained on mutation and metadata features. Deep learning base-lines included CNN, LSTM, transformer, protein language model regression, and HA-only MSA-transformer antigenicity predictors. Scoring baselines tested the effect of using only antigenicity, only dominance, or their direct combination.

Evaluation Metrics

Antigenicity prediction was evaluated using mean absolute error (MAE), root mean squared error (RMSE), Pearson correlation, Spearman rank correlation, and calibration error. MAE and RMSE were calculated as:

$$MAE = \frac{1}{N}\sum_{i=1}^{N} |y_i - \hat{y}_i|, \tag{S35}$$

$$RMSE = \sqrt{\frac{1}{N}\sum_{i=1}^{N} (y_i - \hat{y}_i)^2}. \tag{S36}$$

Future dominance prediction was evaluated using KL divergence:

$$KL(P||Q) = \sum_i p_i \log \frac{p_i}{q_i}, \tag{S37}$$

where P denotes the observed dominance distribution and Q denotes the predicted dominance distribution. Top-k dominant strain recall was calculated as:

$$Recall@k = \frac{|TopK(P) \cap TopK(Q)|}{k}. \tag{S38}$$

Vaccine ranking was evaluated using season-wise empirical normalized coverage score, mean rank of the selected candidate, best-tested-strain rate, and win rate:

$$WinRate = \frac{\text{Number of seasons where proposed selection outperforms baseline}}{\text{Total seasons}}. \tag{S39}$$

Correlation analysis used Pearson correlation for approximately normal data and Spearman rank correlation when monotonic association was more appropriate. Statistical comparisons used paired Wilcoxon signed-rank tests across seasons, and multiple comparisons were controlled with the Benjamini–Hochberg false discovery rate procedure. Calibration was evaluated using expected calibration error, 95% interval coverage, negative log-likelihood, and reliability diagrams.

Implementation Details

All models were implemented in Python using PyTorch for neural network training and scikit-learn for conventional machine learning baselines. XGBoost was used for gradient-boosted tree baselines. Neural models were optimized with AdamW. The temporal module used a Dormand–Prince solver on a monthly time grid. Early stopping was applied using validation loss, and all rolling-season evaluations used cutoff-restricted training data.

Supplementary Data and Protocol Tables

Supplementary Tables S1–S3 summarize sequence preprocessing, HI-pair construction, and structure-graph mapping. Supplementary Table S4 reports the complete rolling evaluation schedule. Supplementary Tables S5 and S6 provide the baseline specifications and implementation settings, respectively.

Table S1. Summary of influenza sequence data after preprocessing. The historical A/H1N1 corpus includes pre-2009 seasonal A/H1N1 and A(H1N1)pdm09 records.

Subtype	Protein	Raw sequences	Retained near-full-length sequences	Non-repeated / unique sequences	Time span	Regions	Main filtering reason
A/H1N1	HA	181,740	152,380	23,736	2003–2023	6	Non-human host, short length, missing collection date, repeated HA sequence
A/H1N1	NA	139,820	112,640	18,920	2003–2023	6	Missing matched HA/NA pair, incomplete NA length, missing date
A/H3N2	HA	212,350	169,740	28,546	2003–2023	6	Non-human host, short length, missing collection date, repeated HA sequence
A/H3N2	NA	156,430	124,980	21,860	2003–2023	6	Missing matched HA/NA pair, incomplete NA length, missing date

Note: The 2003–2023 A/H1N1 counts combine historical pre-2009 seasonal A/H1N1 and pandemic-derived A(H1N1)pdm09 records. The two groups were distinguished using collection date and available lineage or clade metadata. Rolling target-season evaluation from 2012–2013 onward concerns A(H1N1)pdm09.

Table S2. Summary of HI antigenicity data used for vaccine–virus pair learning. The A/H1N1 counts include available pre-2009 seasonal A/H1N1 and A(H1N1)pdm09 vaccine–virus pairs.

Subtype	Virus sequences	Vaccine sequences	Processed HA HI pairs	HA–NA matched HI pairs	Time span	Excluded strain-name rate
A/H1N1	3,068	109	70,631	58,420	2003–2023	20.0% virus; 5.0% vaccine
A/H3N2	2,731	255	63,299	51,870	2003–2023	26.0% virus; 11.0% vaccine

Note: A/H1N1 antigenicity metrics were estimated from the available combined historical H1N1 HI corpus. Target-season dominance and vaccine-ranking evaluation from 2012–2013 onward concerns A(H1N1)pdm09.

Table S3. Protein structure resources and residue-contact graph construction.

Subtype	Protein	Representative experimental templates	Sequence-mapped contact graphs	Mean nodes	Mean edges	Contact threshold
A/H1N1	HA	1	23,736	553	5,740	8 Å
A/H1N1	NA	1	18,920	469	4,580	8 Å
A/H3N2	HA	1	28,546	566	5,910	8 Å
A/H3N2	NA	1	21,860	470	4,620	8 Å

Table S4. Rolling retrospective evaluation design. The cutoff uses only data collected before 1 February of the target year for model training.

Target season	Training cutoff	Evaluation window	Subtype	Candidate window	Evaluated candidate set	Evaluation labels
2012–2013	2012-02-01	2012-10 to 2013-03	A/H1N1	Previous 36 months	50-strain eligible set	HI, dominance, empirical NCS
2012–2013	2012-02-01	2012-10 to 2013-03	A/H3N2	Previous 36 months	51-strain eligible set	HI, dominance, empirical NCS
2013–2014	2013-02-01	2013-10 to 2014-03	A/H1N1	Previous 36 months	50-strain eligible set	HI, dominance, empirical NCS
2013–2014	2013-02-01	2013-10 to 2014-03	A/H3N2	Previous 36 months	51-strain eligible set	HI, dominance, empirical NCS
2014–2015	2014-02-01	2014-10 to 2015-03	A/H1N1	Previous 36 months	50-strain eligible set	HI, dominance, empirical NCS
2014–2015	2014-02-01	2014-10 to 2015-03	A/H3N2	Previous 36 months	51-strain eligible set	HI, dominance, empirical NCS
2015–2016	2015-02-01	2015-10 to 2016-03	A/H1N1	Previous 36 months	50-strain eligible set	HI, dominance, empirical NCS
2015–2016	2015-02-01	2015-10 to 2016-03	A/H3N2	Previous 36 months	51-strain eligible set	HI, dominance, empirical NCS
2016–2017	2016-02-01	2016-10 to 2017-03	A/H1N1	Previous 36 months	50-strain eligible set	HI, dominance, empirical NCS
2016–2017	2016-02-01	2016-10 to 2017-03	A/H3N2	Previous 36 months	51-strain eligible set	HI, dominance, empirical NCS
2017–2018	2017-02-01	2017-10 to 2018-03	A/H1N1	Previous 36 months	50-strain eligible set	HI, dominance, empirical NCS
2017–2018	2017-02-01	2017-10 to 2018-03	A/H3N2	Previous 36 months	51-strain eligible set	HI, dominance, empirical NCS
2018–2019	2018-02-01	2018-10 to 2019-03	A/H1N1	Previous 36 months	50-strain eligible set	HI, dominance, empirical NCS
2018–2019	2018-02-01	2018-10 to 2019-03	A/H3N2	Previous 36 months	51-strain eligible set	HI, dominance, empirical NCS
2019–2020	2019-02-01	2019-10 to 2020-03	A/H1N1	Previous 36 months	50-strain eligible set	HI, dominance, empirical NCS
2019–2020	2019-02-01	2019-10 to 2020-03	A/H3N2	Previous 36 months	51-strain eligible set	HI, dominance, empirical NCS
2020–2021	2020-02-01	2020-10 to 2021-03	A/H1N1	Previous 36 months	Strain-ranking only; excluded from VE correlation	HI, dominance, empirical NCS
2020–2021	2020-02-01	2020-10 to 2021-03	A/H3N2	Previous 36 months	Strain-ranking only; excluded from VE correlation	HI, dominance, empirical NCS
2021–2022	2021-02-01	2021-10 to 2022-03	A/H1N1	Previous 36 months	50-strain eligible set	HI, dominance, empirical NCS
2021–2022	2021-02-01	2021-10 to 2022-03	A/H3N2	Previous 36 months	51-strain eligible set	HI, dominance, empirical NCS

Table S5. Baseline models and evaluation purpose.

Baseline	Input features	Main output	Purpose of comparison	Expected limitation
Last-season dominance	Previous season frequency	Future dominance	Simple temporal baseline	Cannot model antigenic match
Clade-frequency model	Clade frequency trend	Candidate rank	Classical surveillance-based comparison	Coarse clade-level resolution
Phylogenetic ranking	Tree growth and clade position	Candidate rank	Evolutionary comparator	Limited antigenicity learning
Random forest	Sequence-derived mutation features	HI/dominance	Conventional ML baseline	Weak sequence-context learning
XGBoost	Mutation and metadata features	HI/dominance	Strong tabular ML baseline	No direct residue attention
CNN	One-hot sequence representation	HI prediction	Deep sequence baseline	Limited long-range context
LSTM	Sequential amino acid representation	HI/dominance	Recurrent sequence baseline	Weaker long-range modeling than transformer
Transformer	HA/NA tokens	HI/dominance	Standard attention baseline	No structure or diffusion module
Protein language model + regression	Pretrained sequence embeddings	HI/dominance	Foundation-model baseline	Limited task-specific fusion
HA-only MSA-transformer antigenicity model	Aligned pairwise sequences	HI prediction	Strong antigenicity baseline	No future dominance ranking
Dominance-only score	Predicted dominance	Candidate rank	Tests need for antigenicity	Ignores vaccine–virus match
Antigenicity-only score	Predicted HI similarity	Candidate rank	Tests need for future dominance	Ignores future prevalence
FluEvoFormer	HA/NA, structure, time, metadata, HI	Robust vaccine rank	Proposed method	Higher computational cost; requires external validation

Table S6. Implementation and hyperparameter settings.

Component	Setting	Value
Dual HA–NA transformer	Number of layers	12
Dual HA–NA transformer	Hidden dimension	512
Dual HA–NA transformer	Attention heads	8
Dual HA–NA transformer	Dropout	0.10
Graph transformer	Contact threshold	8 Å
Graph transformer	Number of layers	3
Temporal module	ODE solver / time step	Dormand–Prince solver; monthly time grid
Diffusion module	Number of diffusion steps	100
Optimization	Optimizer	AdamW
Optimization	Learning rate	1×10^{-5} to 2×10^{-5}
Optimization	Batch size	16–32 with gradient accumulation
Training	Epochs / steps	100 epochs for dominance; 150,000 steps for antigenicity fine-tuning; early stopping on validation loss
Hardware	GPU / memory	1 NVIDIA A100 GPU, 40 GB memory

Table S7. Candidate-code definitions for coded historical and FluEvoFormer-ranked vaccine strains.

Code pattern	Subtype	Selection type	Definition and reporting basis
Rec-H1N1-Y	A/H1N1	Historical recommendation	Historical A/H1N1 vaccine component recommended for the Northern Hemisphere season beginning in year Y, based on WHO vaccine composition records.
Rec-H3N2-Y	A/H3N2	Historical recommendation	Historical A/H3N2 vaccine component recommended for the Northern Hemisphere season beginning in year Y, based on WHO vaccine composition records.
FEF-H1N1-Y-A	A/H1N1	FluEvoFormer ranked candidate	Highest-ranked eligible A/H1N1 candidate selected by FluEvoFormer from the cutoff-restricted candidate window for the Northern Hemisphere season beginning in year Y. Candidate eligibility used subtype consistency, sequence quality, collection time, and sufficient retrospective HI coverage.
FEF-H3N2-Y-A	A/H3N2	FluEvoFormer ranked candidate	Highest-ranked eligible A/H3N2 candidate selected by FluEvoFormer from the cutoff-restricted candidate window for the Northern Hemisphere season beginning in year Y. Candidate eligibility used subtype consistency, sequence quality, collection time, and sufficient retrospective HI coverage.
FEF-H3N2-2019-A	A/H3N2	FluEvoFormer ranked candidate	Model-ranked A/H3N2 candidate for the 2019–2020 case study. The candidate showed broader predicted clade-level coverage across 3C.2a1b.1a/b and 3C.2a1b.2b/a clusters than the historical recommendation in the retrospective analysis.

Supplementary Computational-Efficiency Figure

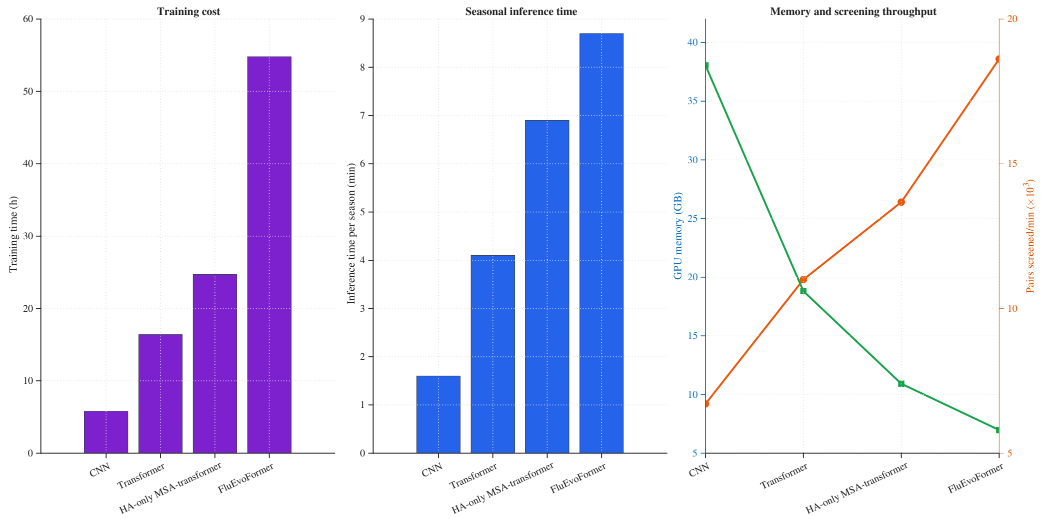

Figure S4. Computational-efficiency comparison of the CNN, transformer, HA-only MSA-transformer, and FluEvoFormer models. The four panels report total training time, inference time per target season, peak GPU memory use, and the number of vaccine–virus pairs screened per minute. Lower training time, inference time, and memory use indicate lower computational cost, whereas higher screening throughput indicates faster candidate evaluation. All neural-model measurements were obtained under the implementation conditions reported in Supplementary Table S6.
